# Wooden steps to shallow depths: A new bathymodiolin mussel, *Vadumodiolus teredinicola*, inhabits shipworm burrows in an ancient submarine forest

**DOI:** 10.1016/j.dsr.2023.104220

**Published:** 2024-01-09

**Authors:** Marvin A. Altamia, Hannah J. Appiah-Madson, Rosalia Falco-Poulin, Bruno Huettel, Maxim Rubin-Blum, Nicole Dubilier, Harald R. Gruber-Vodicka, Nikolaus Leisch, Daniel L. Distel

**Affiliations:** a Ocean Genome Legacy Center, Department of Marine and Environmental Science, Northeastern University, Nahant, MA, USA; b Max Planck Genome Centre Cologne, Max Planck Institute for Plant Breeding Research, Cologne, Germany; c Israel Oceanographic and Limnological Research Institute– IOLR, Haifa, Israel; d Max Planck Institute for Marine Microbiology, Bremen, Germany; e Marine Symbiosis Group, Zoological Institute, Christian-Albrechts-University, Kiel, Germany; f EMBL Heidelberg, Heidelberg, Germany

**Keywords:** Bathymodiolinae, Bivalve evolution, Chemoautotrophic symbiosis, Mytilidae, Teredinidae, Thioautotrophic symbionts

## Abstract

Large mussels of the mytilid subfamily Bathymodiolinae are common inhabitants of deep-sea hydrothermal vents and cold seeps, where gill-borne symbionts allow them to utilize energy-rich compounds such as hydrogen sulfide and methane to support abundant growth. This subfamily also includes smaller symbiont-bearing mussels found on deep-sea wood and organic deposits. Phylogenetic analyses suggest that wood association is ancestral to bathymodiolin evolution. This observation led to the “wooden steps” hypothesis, which proposed that wood and other large organic deposits have acted as evolutionary steppingstones, introducing the progenitors of the modern vent and seep Bathymodiolinae to their remote environments. Although this hypothesis implies an evolutionary trajectory from shallow to deep water, no bathymodiolin species that grows and reproduces at depths less than 100 m has yet been formally described. Here we describe a new bathymodiolin genus and species, *Vadumodiolus teredinicola,* found growing and reproducing at a depth of 18 m in uninhabited shipworm burrows in the remnants of an ancient submerged bald cypress forest off the coast of Alabama. These results demonstrate that the bathymodiolin radiation has not been limited to deep water and that specific association with wood has led to the successful invasion of both deep and shallow marine environments.

## 1. Introduction

Mussels of the subfamily Bathymodiolinae Kenk & B. R. Wilson, 1985 are among the most common inhabitants of deep-sea hydrothermal vents and cold seeps and were among the first marine invertebrates recognized to harbor chemoautotrophic (Cavanaugh, 1983) and methanotrophic (Cavanaugh et al., 1987; Childress et al., 1986) symbionts. This subfamily was erected to accommodate *Bathymodiolus thermophilus* Kenk & B. R. Wilson, 1985, a species of chemoautotrophic mussel discovered at the Galapagos Rift deep-sea hydrothermal vents (Kenk and Wilson, 1985). Since then, over 50 species of Bathymodiolinae have been described and named (McCowin et al., 2020). These species are currently assigned to nine genera that are accepted by the World Register of Marine Species (WoRMS) (Horton et al., 2021): *Adipicola* Dautzenberg, 1927, *Bathymodiolus* Kenk and Wilson, 1985, *Benthomodiolus* Dell, 1987, *Gigantidas* von Cosel and Marshall, 2003, *Idas* Jeffreys, 1876, *Nypamodiolus* Y.-T. Lin, Kiel, T. Xu & J.-W. Qiu, 2022, *Tamu* Gustafson, R. D. Turner, Lutz & Vrijenhoek, 1998, *Terua* Dall, Bartsch & Rehder, 1938, and *Vulcanidas* von Cosel and Marshall, 2003. At least two additional genera have been proposed but not formally described (Samadi et al., 2015; Thubaut et al., 2013). All but one of these species are thought to harbor bacterial ecto- or endosymbionts (Rodrigues et al., 2015), which may include thioautotrophic, methanotrophic, and heterotrophic bacteria (Duperron and Gros, 2016; Duperron et al., 2008a; Laming et al., 2018; Rodrigues et al., 2015).

Although the first species assigned to Bathymodiolinae were exclusively associated with hydrothermal vents and cold seeps, later molecular phylogenetic analyses demonstrated that a group of smaller mussel species often associated with wood, bone, and organic deposits in the deep sea are also members of this subfamily (Distel et al., 2000). At the time, one of these small mussels, *Idas washingtonius* (F. R. Bernard, 1978), had already been shown to harbor sulfur-oxidizing chemoautotrophic symbionts (Deming et al., 1997) and was proposed to utilize hydrogen sulfide produced by the microbial decay of organic matter rather than by hydrothermal vents or other geological sources. These findings suggested that the most recent common ancestor of extant Bathymodiolinae may have been a chemoautotrophic mussel associated with wood and organic matter and that sinking wood, bone, and large organic deposits may have acted as steppingstones that helped to introduce the symbiont-bearing progenitors of modern Bathymodiolinae to deep-sea hydrothermal vents and cold seeps. This hypothesis has come to be known as the “wooden steps to hydrothermal vents” or simply the “wooden steps” hypothesis (Jones et al., 2005; Lorion et al., 2009, 2010, 2013; Miyazaki et al., 2010; Samadi et al., 2007; Thubaut et al., 2013; Zvi-Kedem et al., 2023).

Subsequent studies have supported and extended the wooden steps hypothesis, including phylogenetic analyses employing multiple inference methods and analyzing diverse taxa and gene sets. These studies (i) confirmed the monophyly of Bathymodiolinae including both vent and seep taxa as well as taxa associated with organic remains (Gaudron et al., 2010; Lorion et al., 2009; Samadi et al., 2007; Thubaut et al., 2013), (ii) indicated that the progenitors of modern Bathymodiolinae were likely associated with wood or other types of organic deposits (Lorion et al., 2010, 2013; Samadi et al., 2007; Thubaut et al., 2013), and (iii) suggested that the introduction of Bathymodiolinae from organic deposits to vents and seeps likely occurred in multiple independent events (Lorion et al., 2010, 2013; Thubaut et al., 2013).

Although vents, seeps, sunken wood, and other large organic deposits support the growth of bathymodiolin taxa in the deep sea, there is little evidence that bathymodiolin mussels routinely thrive in shallow-water marine environments. Indeed, the known distribution of Bathymodiolinae is primarily restricted to depths greater than 100 m (Lorion et al., 2010). For example, 6,141 occurrences of Bathymodiolinae were recorded in the Ocean Biogeographic Information System (OBIS, www.obis.org) as of 2023-05-17. Of these, ~99%, 94%, and 84% occurred at minimum depths greater than 100 m, 300 m, and 500 m, respectively. While OBIS includes 67 records of bathymodiolin occurrences at depths of 20 m or less (including 28 records of the species described here and submitted by our authors), these occurrences are only provisionally identified to subfamily or to the genus *Idas,* which has often served as a catch-all for undescribed small bathymodiolins. None have been described in peer-reviewed literature.

Such incompletely documented shallow observations may be misleading. For example, the remains of a bathymodiolin mussel, now known as *Adipicola pelagica* (Forbes, 1854), was first reported on floating whale blubber, suggesting a shallow depth distribution (Woodward, 1854). However, these remains are now thought to have floated to the surface from carcasses on the sea floor (Smith and Baco, 2003), as this species is primarily found at depths of 400–1800 m (Dell, 1987). To our knowledge, no bathymodiolin species described in peer-reviewed literature has been shown to grow and reproduce at depths less than 100 m.

Here we describe *Vadumodiolus teredinicola,* a new genus and species of bathymodiolin mussel found growing and reproducing at depths of 18–20 m in uninhabited burrows of wood-boring and wood-eating bivalves of the family Teredinidae (shipworms). These burrows were formed by shipworms feeding on the submerged wood remnants of an ancient bald cypress (*Taxodium distichum*) forest discovered approximately 13 km off the coast of Orange Beach, Alabama, USA. The site, which is hereafter referred to as the Alabama Undersea Forest (AUF), includes numerous large, exposed stumps of bald cypress trees that remain rooted in life position on the sea floor, as well as an abundance of fallen trunks and partially exposed limbs and branches in what appears to be an ancient riverbank. It has been proposed that the forest was buried by sediments and submerged by sea level rise sometime in the Late Pleistocene, between 72,000 and 45,000 years before present, and was only recently uncovered by storm wave activity (Gonzalez et al., 2017; Reese et al., 2018). Despite having been buried for tens of thousands of years, the recently exposed wood is sufficiently well-preserved to support the growth of large numbers of shipworms. These burrows persist long after the shipworms perish, providing a source of shelter for a variety of organisms, including *V. teredinicola.*

## 2. Methods

### 2.1. Specimen collection

All specimens of *V. teredinicola* examined in this study were collected from found wood or wood collection panels deployed at the AUF site, approximately 13 km off the coast of Orange Beach, AL, USA, at depths ranging from 18 to 20 m. Collection panels were deployed within the bounds of the AUF site in close proximity to exposed natural wood for periods ranging from two months to one year. Each panel included two pieces of dried bald cypress lumber (approximately 17 × 17 × 31 cm and 5 × 20 × 31 cm, respectively) and one dried roughhewn bald cypress log (approximately 17 cm diameter × 31 cm length) tethered together by a braided polyethylene line and attached to a stainless-steel rod embedded in the sea floor (Supplementary Figure SF1). The wood was free to float due to its natural buoyancy and to settle on the seafloor as it became waterlogged. Found wood and collection panels were recovered by hand by SCUBA divers operating from the R/V *E.O. Wilson* between June 2020 and May 2023 and transported to the Dauphin Island Sea Lab, Dauphin Island, AL, USA, and then to the Marine Science Center, Northeastern University, Nahant, MA, USA for subsequent examination and dissection. Type specimens were deposited at the Museum of Comparative Zoology, Harvard University, under the accession numbers (MCZ: Mala:399936–399939). Non-type materials were deposited to the Ocean Genome Legacy Collection (Falco et al., 2022) at Northeastern University under the accession numbers OGL:Genomic:29888, 31606–31614, 31915–31918, 31982, 31985, 32007, 32013–32014, 32016–32024, 35135–35138, 35151–35160, 35163, 35171, 35179, 35863–35878, 36415–36420, and 36436 (Ocean Genome Legacy, 2023).

### 2.2. DNA extraction, metagenome sequencing, and assembly

For metagenome sequencing, three specimens of *V. teredinicola* were dissected while submerged in RNA*later* (ThermoFisher Scientific, Waltham, MA, USA) and DNA was extracted using the DNeasy Blood and Tissue kit (QIAGEN, Hilden, Germany) following the manufacturer’s instructions with the following amendments: incubation time in the initial lysis buffer was extended to 24 h at 56 °C, DNA was eluted into 40 μl of elution buffer pre-warmed to 37 °C, and the eluent was used to elute a second time to increase DNA yield. DNA was stored at 4 °C until further processing.

Genomic DNA quality and quantity were assessed using the Agilent Femto pulse (Agilent Technologies, Santa Clara, CA, USA), and concentration was adjusted as necessary by magnetic bead binding (AMPure, Beckman-Coulter, Pasadena, CA, USA) followed by elution in an appropriate volume. Tn5 transposase-based libraries (Rowan et al., 2019) were generated from 1 ng of genomic DNA on a Sciclone G3 robotic liquid handling device (PerkinElmer, Waltham, MA, USA). Library size distribution and quality were assessed on a LabChip Touch nucleic acid analyzer (PerkinElmer, Waltham, MA, USA) and quantified by fluorometry. Libraries were sequenced on an Illumina HiSeq 3000 (Illumina, San Diego, CA, USA) in 2 × 150 bp paired-end read mode.

The quality of raw Illumina metagenome reads was evaluated using FastQC (Andrews, 2010) and FastP (Chen et al., 2018). Pair-ended reads for each tissue sample were assembled in SPADES v3.15 (Bankevich et al., 2012) using kmers 21, 41, 61, 81, 101, and 121. The assembled metagenomic contigs were then binned using metaBAT2 (Kang et al., 2019). CheckM lineage_wf (Parks et al., 2015) was used to determine which bins were of bacterial origin and to assess the completeness of each metagenomic bin. To collect additional bacterial contigs shorter than 1.5 kbp that were not binned by metaBAT2 from assembled metagenomes, we performed BLAST (Altschul et al., 1990) nucleotide searches with an e-value cutoff of 1 × 10^−50^ against the assembled metagenome using the 16S and 23S rRNA genes, and coding sequences of *Bathymodiolus thermophilus* EPR9N (NCBI Accession CP024634) as query. The resulting contigs were merged with metaBAT2 contigs, and duplicates were removed (Rowan et al., 2019). The phylogenetic identity of the binned bacterial contigs, the merged contigs with significant BLAST hits, and a combination of both was determined using GTDB-tk v2.0 (Chaumeil et al., 2019). Genome assemblies were annotated using Prokka (Seemann, 2014) implemented in Galaxy (2022).

### 2.3. Phylogenetic analyses

The *V. teredinicola* nuclear 18S and 28S rRNA genes and the mitochondrial 16S rRNA and *cox*1 genes were identified in the metagenomic assemblies by BLAST searches. Using MAFFT v7 (Katoh and Standley, 2013), these sequences were then aligned with those of representatives of bathymodiolin taxa for which at least three of the four marker genes were available in Genbank as well as selected Modiolinae and Lithophagainae which were included as reference taxa (accession numbers listed in Supplementary Table ST1). The genes were then concatenated in the following order: 18S (1,546 bp), 28S (700 bp), 16S rRNA (342 bp), and *cox*1 (507 bp), resulting in a final alignment length of 3,095 bp. The best nucleotide substitution model for each gene was estimated using jModelTest v2 (Darriba et al., 2012) and corrected using the Akaike Information Criterion (Akaike, 1974). The phylogenetic relationships among *V. teredinicola* and related taxa were determined by Bayesian inference using MrBayes (Ronquist and Huelsenbeck, 2003). A total of 30 million Markov Chain Monte Carlo chains were used, employing the appropriate nucleotide substitution model for each gene partition. The results were subsampled every 2,000 chains, discarding the first 25% as analytical burn-in. A maximum-likelihood-based inference analysis was also performed using iQ-Tree v2.2.0 (Minh et al., 2020). The appropriate nucleotide substitution model for each gene segment was determined using the ModelFinder (Kalyaanamoorthy et al., 2017) module implemented in iQ-Tree. A total of 1,000 ultrafast bootstrap replicates were used to evaluate the support value for each node.

To examine the phylogenetic position of the bacterial marker genes identified in *V. teredinicola* gill metagenomes relative to known symbiotic and free-living thioautotrophic bacteria, a maximum likelihood analysis was performed using RAxML v8.2 (Stamatakis, 2014) with the PROTGAMMABLOSUM62 amino acid substitution model and 1,000 bootstrap replicates on an alignment of concatenated protein sequences encoded by 120 conserved single copy genes identified and aligned using GTDB-tk (Chaumeil et al., 2019). Additionally, small subunit rRNA sequences present in the metagenome were reconstructed and identified phylogenetically using phyloFlash 3.4 (Gruber-Vodicka et al., 2020) and BLAST, and phylogenetic trees were inferred using MrBayes and iQ-Tree.

### 2.4. Microscopy

For microscopy, specimens of *V. teredinicola* were fixed in 4% paraformaldehyde (PFA) in filter-sterilized seawater for 3–5 h at 4 °C. After fixation, the specimens were dehydrated by stepwise incubations in 30%, 50%, and 70% ethanol for 15 minutes at each step. For imaging of the valves and prodissoconchs, specimens were submerged in 70% ethanol and imaged using an AxioZoom v16 microscope (Zeiss, Oberkochen, Germany). Multiple images were taken at various focal planes, and focus stacking was performed in Photoshop v24.0 (Adobe, San Jose, CA) to increase the depth of field at high magnification.

### 2.5. Histology and 16S-rRNA-directed fluorescence in situ hybridization

For histology and 16S-rRNA-directed fluorescence *in situ* hybridization, select PFA-fixed specimens stored in 70% ethanol were rehydrated through successive 15-minute incubations in 50% and 30% ethanol and then transferred to a 5% acetic acid solution and agitated slowly on a rocker platform at room temperature for 5 h to decalcify the valves. Subsequently, specimens were dehydrated through successive 15-minute incubations in 30%, 50%, and 70% ethanol. Specimens were then embedded in paraffin, cut into 5 μm sections, and mounted on glass slides at Beth Israel Deaconess Medical Center Histology Core (Boston, MA, USA). Adjacent slides were used for histochemical staining (hematoxylin and eosin) and 16S rRNA fluorescence *in situ* hybridization. A Cy5-labelled bacterial domain targeted 16S rRNA probe, EUB338 (5′-AGC CAT GCA GCA CCT GTC TC-3′) (Daims et al., 1999) was used for general bacterial detection and a Cy3-labelled probe, Bthio-193 (5′-CGA AGA TCC TCC ACT TTA-3′) (Duperron et al., 2007) was used to specifically detect 16S rRNAs of known bathymodiolin thioautotrophic symbionts in tissue sections. Probes were applied to deparaffinized tissue sections in the presence of 20% formamide, as described in (Fuchs et al., 2007). An antisense probe, EUB338 NON (5′-GAG ACA GGT GCT GCA TGG CT-3′) was applied as a negative control. Slides were mounted using ProLong Glass Antifade Mountant with NucBlue stain (ThermoFisher Scientific, Waltham, MA, USA) and allowed to cure at room temperature for 48 h. Sections were imaged on a Zeiss LSM 880 confocal microscope (Zeiss International, Oberkochen, Germany).

### 2.6. Morphometrics

All specimens of *V. teredinicola* used for morphometrics were removed from a single roughhewn bald cypress log (approximate dimensions: 17 cm diameter × 31 cm length) deployed as part of a collection panel at the AUF site for 8 months. The maximum length and height for each specimen were determined photomorphometrically. Due to the fragility of the valves and the difficulty of standing them on their narrow dorsal or ventral surfaces, the widths of the valves were not determined.

### 2.7. Microcomputed tomography

Microcomputed tomography was used to evaluate three-dimensional relationships among morphological features. A representative PFA-fixed specimen of *V. teredinicola* (6137L-C), stored in 70% ethanol, was transferred to 1% phosphotungstic acid (PTA) in absolute ethanol for 15 days at room temperature to improve the contrast of the soft body parts. The PTA-contrasted specimen was placed in a 0.5 ml microfuge tube, immobilized in 2% low-melt agarose, and imaged using a Zeiss Xradia 520 Versa microCT device (Zeiss, Oberkochen, Germany) housed at the Boston University microCT and X-ray imaging facility (Boston, MA, USA). Briefly, the microfuge tube containing the sample was mounted on a plastic pipet tip and scanned using the following parameters: 60 kV, 5 W, LE1 source filter, 0.4X objective, 2-s exposure time, 3001 projections, at 10 μm resolution (pixel size).

## 3. Results and discussion

### 3.1. Discovery and collection of a new mussel species

On June 22, 2020, a minute mussel, provisionally identified as a new species of the mytilid subfamily Bathymodiolinae, was discovered within an uninhabited shipworm burrow in a Late Pleistocene wood deposit on the sea floor off the coast of Alabama. Over the next 30 months, 124 specimens of this mussel species were collected at the same site, both in naturally occurring wood and in bald cypress collection panels deployed at the site.

**Fig. 1.**
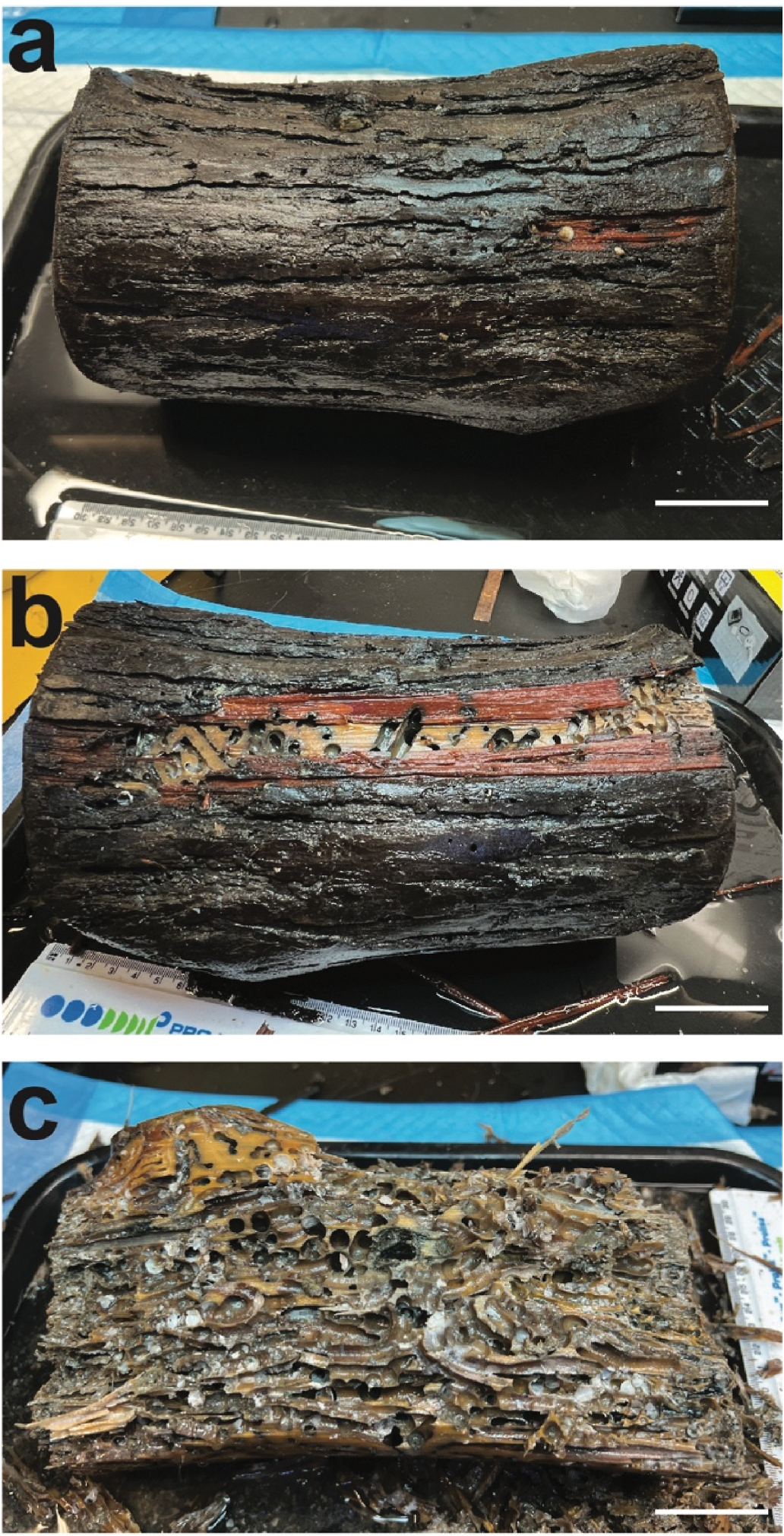
Bald cypress log deployed at the Alabama Undersea Forest site for eight months. (a) Undissected log, (b) log with a portion of the bark removed exposing empty shipworm burrows near wood surface, (c) dissected log exposing crowded shipworm burrows within the interior of the wood. Scale bar, 5 cm.

To obtain specimens and determine growth and recruitment characteristics, collection panels were deployed at the AUF site for intervals ranging from 2 to 12 months. After 2 months, panels were densely populated with living and actively wood-feeding specimens of the shipworm species *Bankia gouldi, Nototeredo knoxi,* and *Teredothyra matocotana*, ranging from 3 mm to 2 cm in length. No mussel specimens were found in these panels. However, numerous specimens of the unidentified mussel were collected in wood panels placed at the site for periods of 8–12 months. This wood appeared intact on the surface (Fig. 1a) but was extensively riddled with shipworm burrows internally (Fig. 1c). Most of these burrows were empty indicating that shipworms had exhausted the available wood and died. Specimens of the new mussel species were found within the empty burrows.

### 3.2. Phylogenetic analyses

**Fig. 2.**
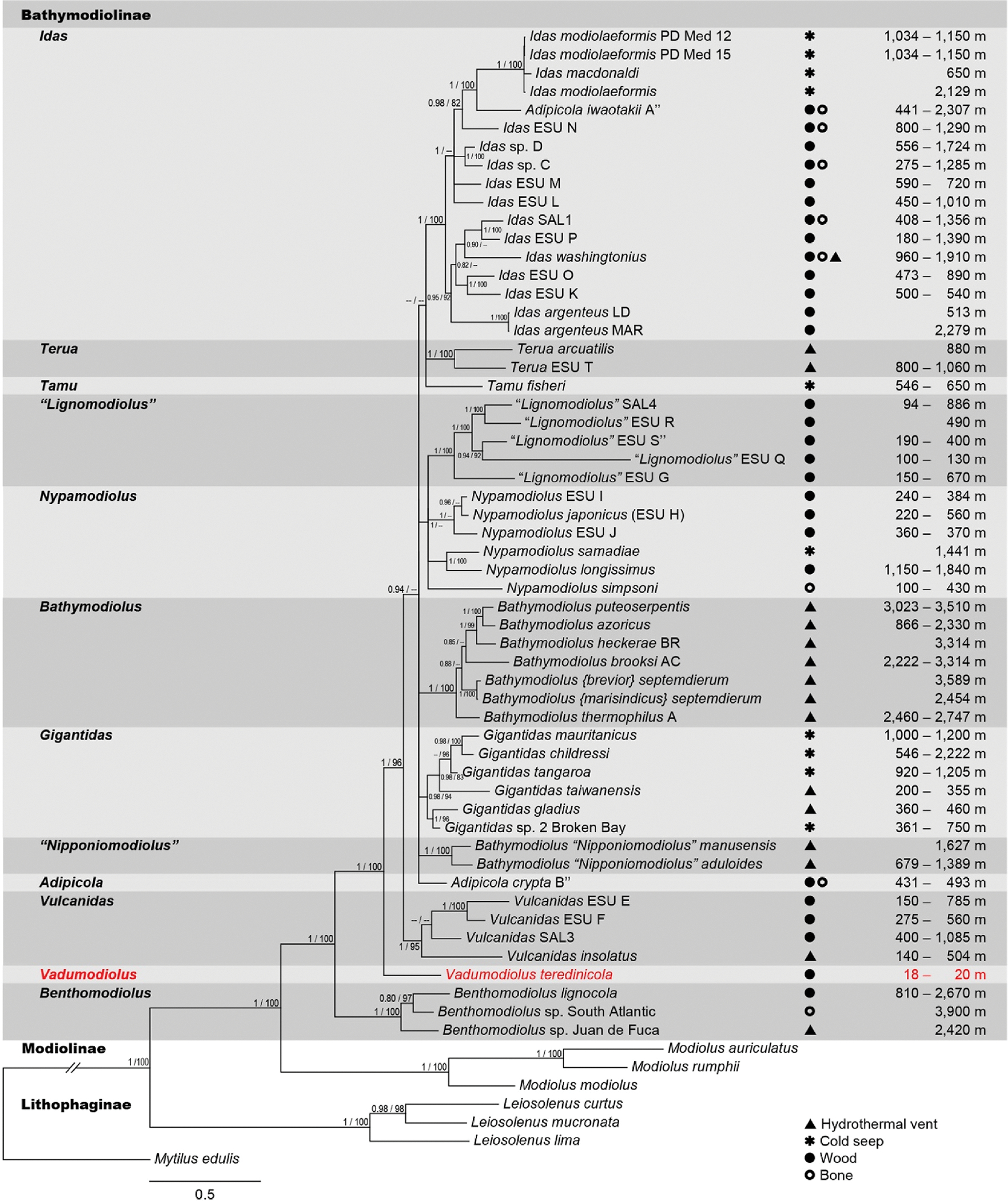
Phylogram depicting inferred phylogenetic relationships between *Vadumodiolus teredinicola* and representative Bathymodiolinae, showing observed habitat type and depth of occurrence. Bayesian inference (MrBayes) was used to create a phylogram based on an alignment of partial sequences of four concatenated gene loci, nuclear 18S rRNA (1,546 bp) and 28S rRNA (700 bp) and mitochondrial 16S rRNA (342 bp) and *cox*1 (507 bp) genes. Taxon selection includes *V. teredinicola*, 54 bathymodiolin species from hydrothermal vents, cold seeps, and organic deposits, and 7 non-bathymodiolin reference taxa (GenBank accession numbers are in Supplementary Table ST1). Posterior probabilities greater than 0.90 are displayed at each associated node to the left of the slash. To the right of the slash are bootstrap proportions of 1,000 replicates for nodes also supported by maximum likelihood analysis (iQ-Tree) of the same sequence alignment. Scale bar, 0.1 substitutions per 100 base pairs. Depth ranges and habitat types for *V. teredinicola* are from this study, others are from (Thubaut et al., 2013; Zvi-Kedem et al., 2023). The generic names “*Lignomodiolus*” proposed by (Thubaut et al., 2013) and “*Nipponiomodiolus*” proposed by (Samadi et al., 2015) correspond to clades L8 and L6 of (Lorion et al., 2013) respectively, and are nomen nuda as they have not been formally described.

To determine the phylogenetic affiliation of the newly collected mussels, Bayesian and maximum likelihood analyses were performed based on partial sequences of four genetic loci (18S rRNA, 28S rRNA, 16S rRNA, and *cox*1; Fig. 2). These analyses resulted in phylogenetic trees with topologies very similar to those recovered in previous phylogenetic studies of Bathymodiolinae (Lin et al., 2022; Lorion et al., 2013; McCowin et al., 2020; Thubaut et al., 2013; Xu et al., 2019), with significant support for clades corresponding to the subfamily Bathymodilinae, the accepted bathymodiolin genera *Benthomodiolus*, *Gigantidas*, *Terua,* and *Vulcanidas* (Horton et al., 2021), and two proposed genera that are not yet formally described (Samadi et al., 2015; Thubaut et al., 2013). Our results are also consistent with prior studies (Lin et al., 2022; Lorion et al., 2010, 2013; McCowin et al., 2020; Miyazaki et al., 2010; Samadi et al., 2015; Xu et al., 2019), in showing that some accepted bathymodiolin genera, including *Adipicola, Bathymodiolus,* and *Idas,* are not monophyletic, as currently classified in the World Register of Marine Species accessed on 2023-05-18 (Horton et al., 2021).

The analyses presented here strongly support the assignment of the new mussel species to the mytilid subfamily Bathymodiolinae. Consistent with previous studies (Laming et al., 2018; Lin et al., 2022; Lorion et al., 2013; McCowin et al., 2020; Thubaut et al., 2013), our analyses indicate that *Benthomodiolus* forms the deepest branch of the bathymodiolin radiation and is sister to all remaining Bathymodiolinae. The new mussel species appears on a well-supported branch of the bathymodiolin tree that falls between *Benthomodiolus* and *Vulcanidas*, indicating that progenitors of this species emerged early in bathymodiolin evolution. The phylogenetic position of these newly collected mussels, their evolutionary distance from other Bathymodiolinae, and the morphological features and habitat preferences to be described herein preclude their assignment to currently accepted and proposed genera. For these reasons, we propose the new genus and species *Vadumodiolus teredinicola*.

### 3.3. Description of the new genus

Class Bivalvia Linnaeus, 1758

Order Mytilida Férussac, 1822

Family Mytilidae Rafinesque, 1815

Subfamily Bathymodiolinae Kenk & B. R. Wilson, 1985

*Vadumodiolus* n. gen.

http://zoobank.org/NomenclaturalActs/859907CA-061A-4DCE-85AA-197CB8BB16E1.

#### 3.3.1. Type and only species

*Vadumodiolus teredinicola* n. gen. n. sp.

#### 3.3.2. Diagnosis

Shells small, ovoid, elongate, equivalve, extremely thin, transparent, very fragile, dorsal margin slightly convex, ventral margin slightly concave, anterior and posterior margins rounded, widest and tallest near the posterior end of the umbo, tapering smoothly in height and width toward the posterior end, strongly inequilateral with umbones very near the anterior margin but not terminal. The posterior pedal-byssal musculature is formed of right and left sets of muscle bundles, each comprised of four thin muscular strands that are similar in diameter and run in parallel as a single group, not divided into anterior and posterior groups of bundles, from their shell attachment near the posterior adductor and fusing into a single strand very near their attachment to the foot. The right and left anterior pedal retractors are each formed of a single strand that attaches to the shell dorsal to the anterior adductor at the anterior end of the umbonal cavity.

#### 3.3.3. Etymology

*Vadumodiolus* literally means ‘shallow modiolin mussel’ from the Latin *vadum*, a shallow water body, reflecting the shallow depth of the type locality.

#### 3.3.4. Remarks

The shells of *Vadumodiolus* are differentiated from those of *Bathymodiolus, Gigantidas*, *Nypamodiolus*, *Vulcanidas*, and *Tamu* in that they are small, extremely delicate, oblong in shape, tallest near the anteriorly positioned umbones, and decorated with delicate calcified spines. In contrast, the shells of the latter five genera are comparatively large and thick, become tallest toward the posterior, and lack calcified spines (Gustafson et al., 1998; Hashimoto and Furuta, 2007; Hashimoto and Okutani, 1994; Lin et al., 2022; von Cosel and Marshall, 2010; Xu et al., 2019). Additionally, the extreme anterior subterminal position of the umbones of *Vadumodiolus* distinguishes their shells from those of *Benthomodiolus* and *Terua* (Dell, 1987, 1995; Oliver, 2015). Although the shells of *Vadumodiolus teredinicola* are similar in form to those of *Adipicola iwaotakii* and *A*. *crypta* (Dell, 1987), molecular phylogenetic analyses presented here and elsewhere show that these three species are deeply divergent, with *A. iwaotakii* falling in a clade containing numerous species of *Idas,* including *I*. *argenteus,* the type species of the genus. The nonmonophyly of *Idas* and *Adipicola,* along with the small number of shell characters available for comparison and high degree of observed polymorphism (Lin et al., 2022; Lorion et al., 2010), create challenges for identifying shell features that differentiate members of these genera from *Vadumodiolus.* However, to our knowledge, calcified shell spines have not been reported in *Idas* and *Adipicola*, and so may be a distinguishing feature of *Vadumodiolus*.

Characteristics of the gills also differentiate *Vadumodiolus* from *Bathymodiolus*, *Gigantidas*, *Nypamodiolus*, *Vulcanidas*, and *Tamu*. The ctenidia of *Vadumodiolus* are thin and transparent, while those of the other taxa are comparatively thick, fleshy, and opaque (Gustafson et al., 1998; Lin et al., 2022; von Cosel and Marshall, 2010; Xu et al., 2019). Additionally, *Vadumodiolus* is distinct from *Benthomodiolus* in that the outer demibranchs of *Vadumodiolus* are about 20% shorter along the anteroposterior axis than the inner demibranchs, while the inner and outer demibranchs of *Benthomodiolus* are similar in length (Oliver, 2015).

Pedal-byssal musculature is also helpful in differentiating *Vadumodiolus* from other bathymodiolin genera. In *Bathymodiolus*, *Gigantidas*, and *Benthomodiolus* (Gustafson et al., 1998; Oliver, 2015; Xu et al., 2019), the muscle bundles that form the posterior byssal retractors divide into distinct anterior and posterior groups. In contrast, the posterior retractors of *Vadumodiolus* are formed of four muscle bundles that extend as a single narrowly spaced group toward their shell attachments near the posterior adductor. Furthermore, in *Bathymodiolus*, *Gigantidas*, *Nypamodiolus*, and *Vulcanidas*, the posterior byssal retractors and posterior pedal retractors are distinct and have separate attachments to the foot (Gustafson et al., 1998; Lin et al., 2022; von Cosel and Marshall, 2010; Xu et al., 2019). This arrangement differs from that observed in *Vadumodiolus,* where the posterior retractor muscle bundles merge to form a single attachment to the foot. The pedal-byssal musculature of *Vadumodiolus* also differs from that described for *I. argenteus,* which has separate posterior pedal and posterior byssal retractors (Ockelmann and Dinesen, 2011) but is similar to that described for *Adipicola osseocola* (Dell, 1987). Thus, as previously suggested (Lin et al., 2022; Lorion et al., 2010), more comprehensive and coordinated molecular and morphological analyses will be required to reconcile the taxonomy and evolutionary history of Bathymodiolinae and so allow confident identification of morphological characters that differentiate bathymodiolin genera and species.

### 3.4. Description of the new species

*Vadumodiolus teredinicola* n. gen. n. sp.

http://zoobank.org/NomenclaturalActs/72DAEEB3-DAFF-46E9-88BE-C4A89A35F4DA.

#### 3.4.1. Material examined

##### Holotype.

**Fig. 3.**
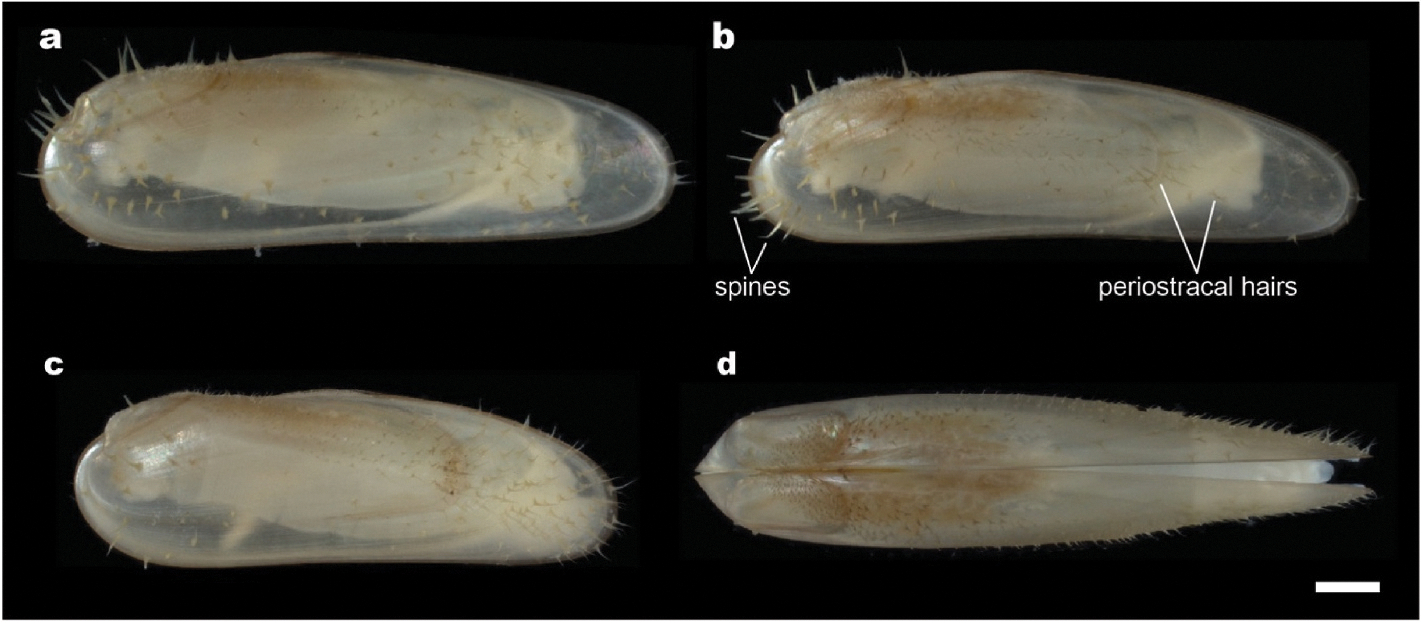
Morphology of *Vadumodiolus teredinicola.* (a) holotype (6317L-B); (b) paratype 6317L-D, (c) paratype (6317L-C), (d) paratype (6317L-A); (a–c) left side view; (d) dorsal view. Scale bar, 1.0 mm.

1 specimen, specimen ID 6317L-B (Fig. 3a) found in a bald cypress log exposed at the type locality for 8 months. Dimensions in mm. Length 9.33; Height 2.58; Width 2.70. *Paratypes*. Include 3 additional specimens (Fig. 3b and d) from the same log. The shells are of similar appearance and proportions as the holotype; dimensions of type and additional non-type materials are reported in Supplementary Table ST5.

#### 3.4.2. Diagnosis

**Fig. 4.**
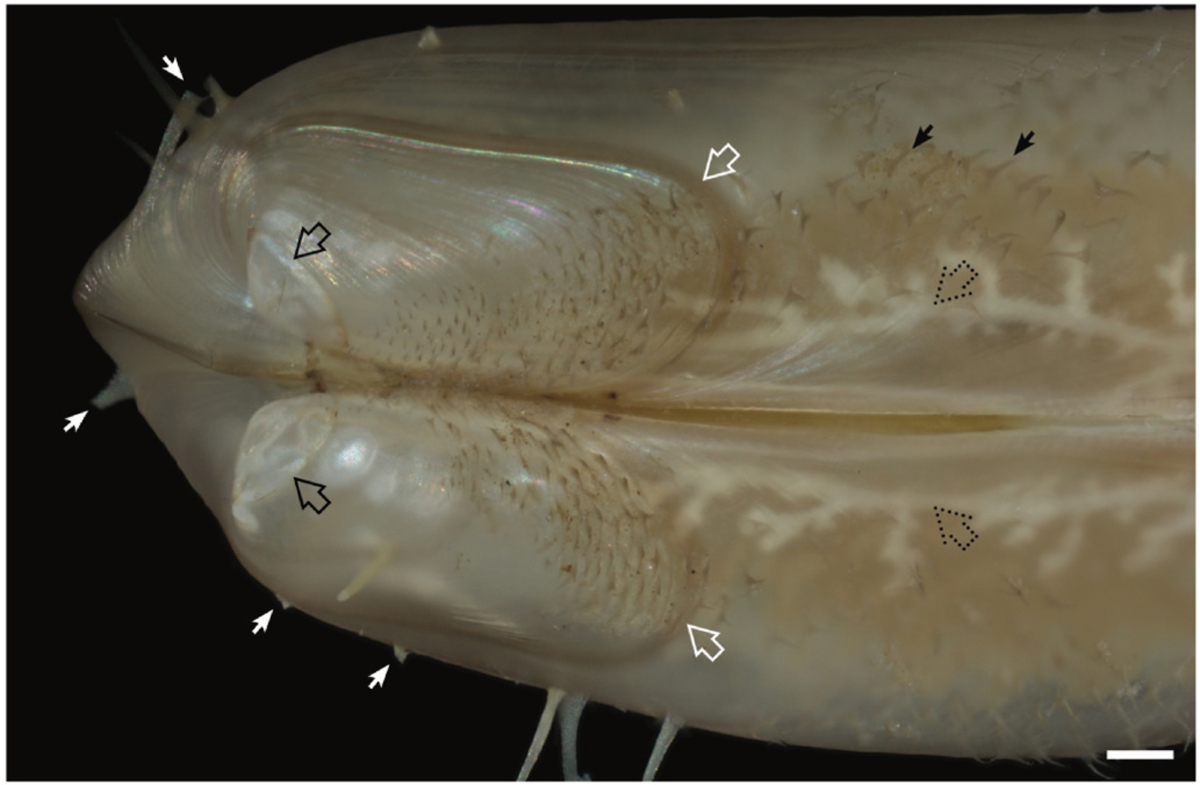
Detail of the valves of *Vadumodiolus teredinicola*. Solid white arrows show the base of fragile calcified spines that were broken during handling; open white arrows show the sharp demarcation that may indicate the transition between the plantigrade and juvenile dissoconch; solid black arrows show the periostracal hairs; open black arrows indicate the edge of the second prodissoconch; dashed black arrows show the seminiferous tubules of the male gonad visible through the transparent shells. Scale bar, 0.2 mm.

**Fig. 5.**
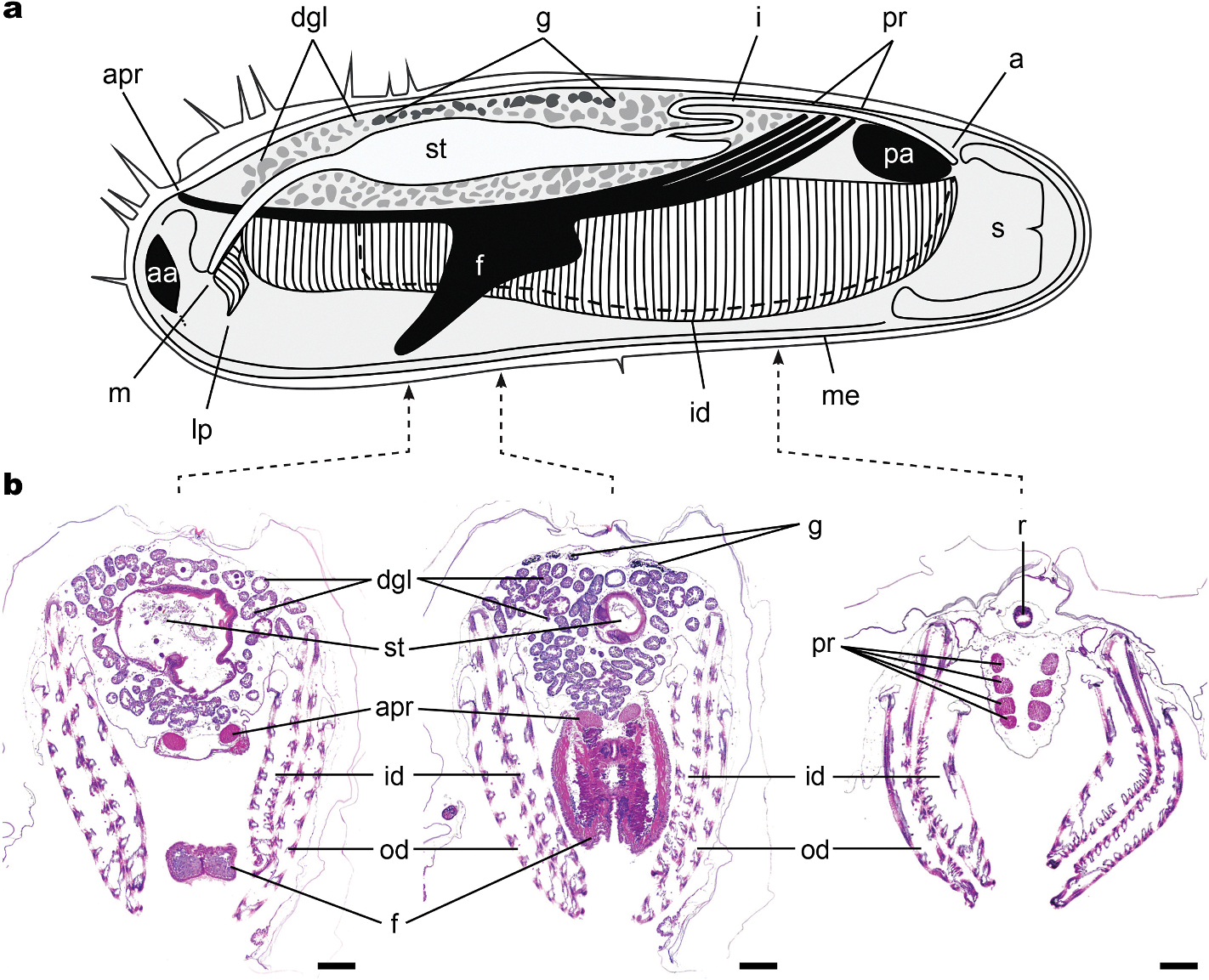
Anatomy of *Vadumodiolus teredinicola*. (a) Diagram depicting a sagittal view along the midline with the left half of the specimen removed. (b–d) Transverse sections from (b) anterior, (c) midbody, and (d) posterior of *V*. *teredinicola* stained with hematoxylin and eocin. a, anus; aa, anterior adductor; apr, anterior pedal retractor; dgl, digestive glands; f, foot; g, gonad; i, intestine; id, inner demibranch; lp, labial palps; m, mouth; me, mantle edge; od, outer demibranch; pa, posterior adductor; pr, posterior retractors; r, rectum; s, siphon; st, stomach. Black, musculature; dark grey, gonadal tissue; medium grey, digestive glands; light grey, mantle tissue. Dashed line in (a) indicates the location of the right outer demibranch, which is obscured from view by the right inner demibranch. Dashed arrows indicate planes of section in (b). Scale bars in (b–d), 0.2 mm.

All features of the genus plus, shell length up to 15 mm and height to 4 mm; highly elongated with an average length-to-height ratio of 3.55 (SD = 0.44*, N* = 21; Supplementary Figure SF2, Supplementary Table ST2). The second prodissoconch is approximately 450 μm in diameter (Fig. 4); umbones are prominent, beaks are at approximately 5% of the shell length from the anterior end, shells near umbones are highly inflated and sharply demarcated from the remainder of the valve; ligament elongate, periostracum translucent, clear to light amber in color, ornamented with long calcified spines that are translucent, very brittle, and very easily broken. These projections, which may be up to 100 μm in width at the base and 500–1,000 μm in length, stand erect, nearly perpendicular to the shell surface and are most prominent on the anterior and posterior ends of the shells. Ctenidia are thin, not fleshy, transparent to white with paired demibranchs of nearly equal height but unequal length; the inner demibranchs extend from the posterior to the anterior adductor; the outer demibranchs terminate anteriorly at approximately 80% of the length of the inner demibranchs. The mantle edge is smooth and free; the ventral gape extends nearly the full length of the shells. The foot is slender and elongate, capable of extending at least one body length beyond the anterior edge of the valves. The distal end of the foot forms a shield- or disk-shaped tip that can grasp both rough and smooth surfaces. Mature male gonads are whitish in color and reticulate in appearance; female gonads are orange in color and globular in appearance. Mature gonads can be readily seen through the transparent shells (Fig. 4, Supplementary Figure SF3). The anatomy of *V. teredinicola* is diagramed in Fig. 5.

#### 3.4.3. Etymology

*teredinicola* comes from the Latin *teredo* ‘shipworm’ and *incola* ‘dweller,’ referring to the fact that the holotype and all specimens collected to date were found within shipworm burrows.

#### 3.4.4. Distribution

*Vadumodiolus teredinicola* is known only from the type locality, approximately 13 km off the coast of Orange Beach, Alabama (30.15 N 87.58 W) at 18–20 m depth in recently exposed remnant wood from an ancient (Late Pleistocene), submerged, bald cypress (*Taxodium distichum*) forest referred to as the Alabama Undersea Forest (AUF). The site lies within the Mississippi, Alabama, Florida (MAFLA) sand sheet, an extensive sandy sea floor region stretching across the northeastern Gulf of Mexico. The ecologically unique and sensitive AUF site is pending protection from commercial exploitation under HR6148: The Alabama Underwater Forest National Marine Sanctuary and Protection Act. The precise coordinates are being withheld until protected status is secured.

### 3.5. Detection and phylogenetic identification of gill symbionts

To search for evidence of bacterial symbionts, we sequenced the gill metagenomes of three specimens of *V. teredinicola* and used a variety of bioinformatic tools, including phyloFlash v3.4 (Gruber-Vodicka et al., 2020), to identify bacterial small subunit (SSU) rRNA gene sequences among the metagenome reads. In the metagenomes, only a single SSU rRNA sequence was detected. In phylogenetic analyses, this sequence formed a well-supported clade with the known sulfur-oxidizing symbionts of bathymodiolin mussels (Supplementary Figure SF4).

**Fig. 6.**
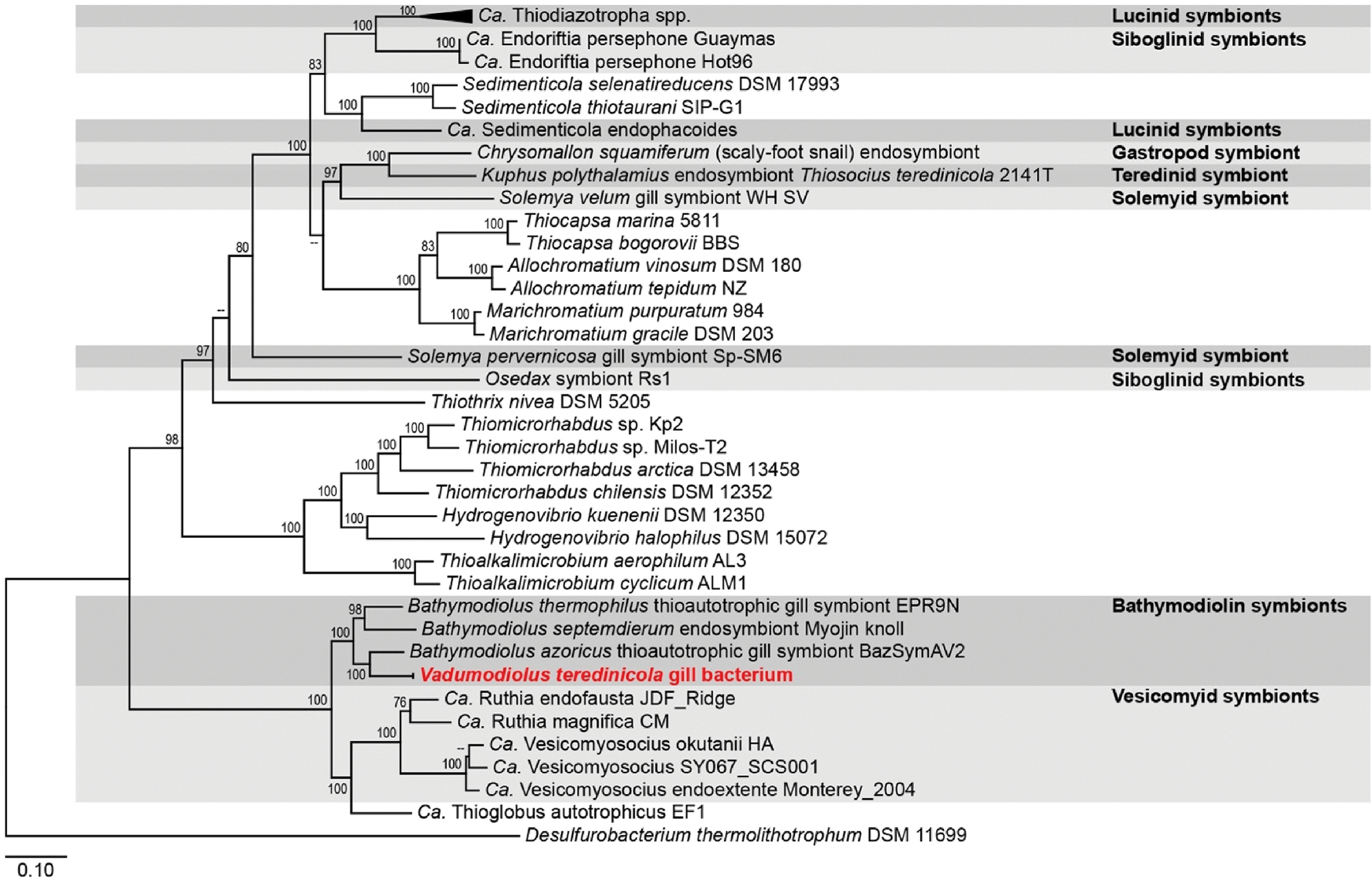
Phylogram depicting inferred phylogenetic relationships between the *Vadumodiolus teredinicola* gill bacterium and representative endosymbiotic and free-living thioautotrophic bacteria. Maximum likelihood analysis (RAxML) was used create a phylogram based on an alignment of sequences from 120 conserved bacterial genes identified in the partial metagenome-assembled genome (MAG) of the *V. teredinicola* gill bacterium and representative thioautotrophic symbionts and free-living bacteria using the Genome Taxonomy Data Base tool kit (GTDB-tk). Bootstrap proportions greater than 70 percent of 1,000 replicates are displayed at associated nodes. Scale bar, substitution rate per site.

Analyses of the full set of reads in gill metagenomes yielded similar results. Although reads of bacterial origin were sparsely represented in each gill metagenome, by combining three gill metagenomes, a single, though highly fragmented, bacterial metagenome-assembled genome (MAG) was constructed (see electronic supplement for additional metagenome assembly details). This single MAG contained 611 contigs and represented approximately 1.06 Mb of genome sequence with 88% completeness as determined by CheckM v.1 (Parks et al., 2015) (Supplementary Table ST3). For comparison, known bathymodiolin symbiont genomes range from 1.47 to 2.8 Mb, indicating that this MAG could encompass 38–72% of a typical bathymodiolin symbiont genome (Supplementary Table ST4). Bayesian and maximum likelihood phylogenies based on an alignment of multiple conserved bacterial genes identified in 40 thioautotrophic bacterial reference species (Supplementary Table ST5) using GTDB-tk (Chaumeil et al., 2019) place the *V. teredinicola* gill bacterium in a well-supported clade with the thioautotrophic symbionts of *Bathymodiolus azoricus, B. septemdierum,* and *B. thermophilus* (Fig. 6, Supplementary Figure SF5). The 2-way genomic average nucleotide identity (gANI) and average amino acid identity (AAI) values computed for *V. teredinicola* and these three bathymodiolin symbionts were 77–82% and 77–81% respectively (Supplementary Figure SF6). These AAI values exceed the mean within-genus similarity calculated for 144 bacterial and archaeal genera (73.9%) (Barco et al., 2020), suggesting that the *V. teredinicola* gill bacterium and these known bathymodiolin symbionts may fall within a single bacterial genus.

**Fig. 7.**
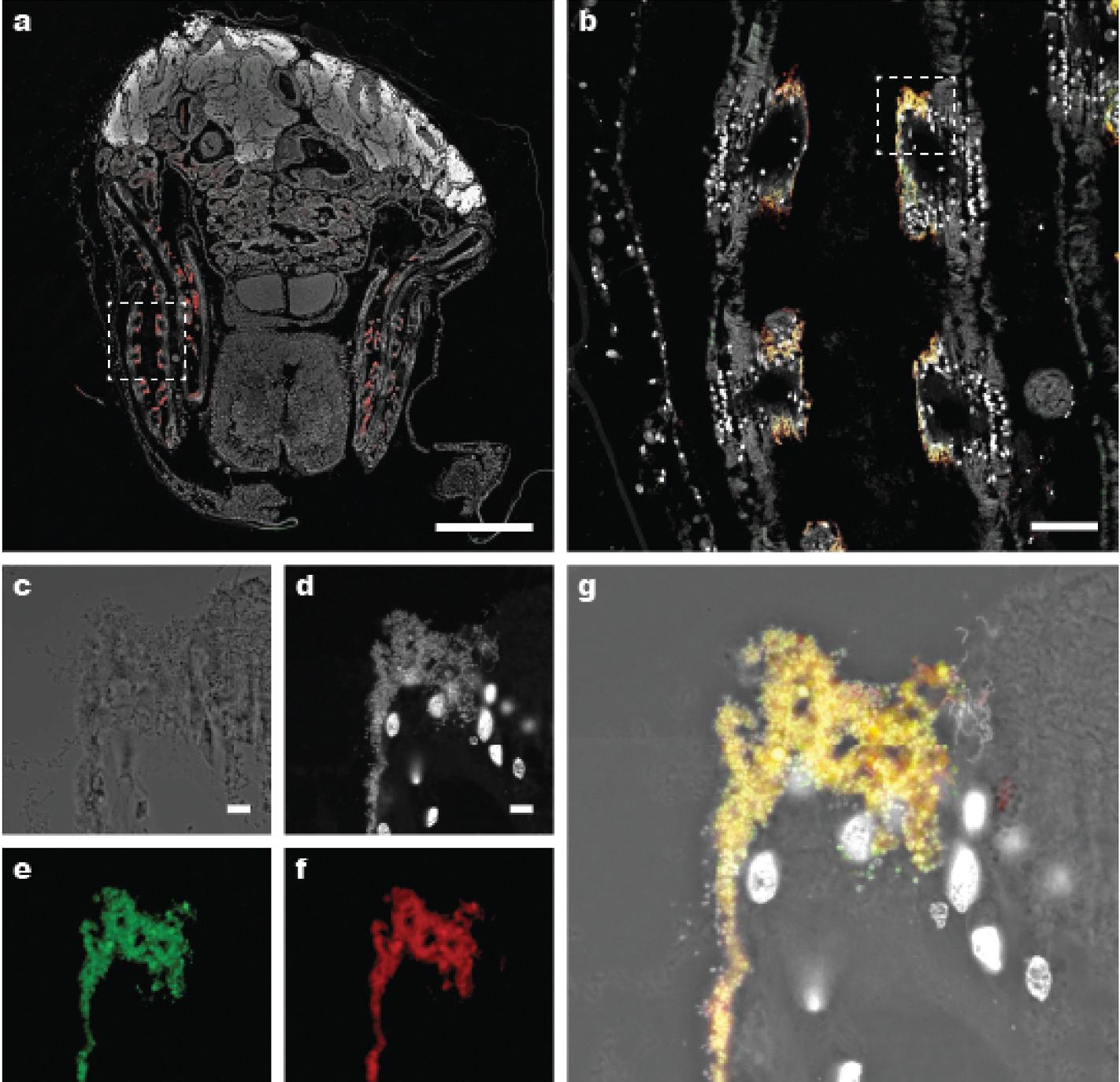
Confocal laser scanning microscopy overview of the distribution of symbionts in *Vadumodiolus teredinicola.* Dual-probe fluorescence *in situ* hybridization (FISH) using both a bacteria-domain-specific 16S rRNA probe Cy5-EUB338 (red) and a probe specifically targeting the 16S rRNAs of known bathymodiolin thioautotrophic symbionts, Cy3-Bthio-193 (green). DNA is stained with NucBlue Nuclear Stain (white). (a) Transverse section through the gill and visceral mass of a mature male specimen of *V. teredinicola*, (b) detail of the boxed region in (a), (c–g) detail of the boxed region in (b). (c) Differential interference contrast image with visible light illumination, (d) NucBlue Nuclear Stain fluorescence channel only, (e) green fluorescence channel only, (f) red fluorescence channel only, and (g) overlay of DIC, NucBlue Nuclear Stain (white), and probes targeting the symbionts (green, Bthio-193; red, EUB338). Note that both EUB338 and Bthio-93 probes (combinatorial colors yellow to orange) hybridized to most bacterial cells within the abfrontal region of the ascending and descending filaments, indicating phylogenetic identity and location similar to the symbionts of other bathymodiolin mussels. Scale bars, (a) 500 μm; (b) 50 μm; (c–g) 5 μm.

Fluorescent *in situ* hybridization (FISH) experiments using a bacteria-domain-specific probe EUB338 (Daims et al., 1999) and a probe specifically targeting the 16S rRNAs of known bathymodiolin thioautotrophic symbionts Bthio-193 (Duperron et al., 2008b) demonstrated that both probes hybridized to bacterial cells detected within the abfrontal region of the ascending and descending filaments (Fig. 7). No hybridization was observed in the gills using antisense versions of the probes (Supplementary Figure SF7). The results of these hybridizations indicate that the gill bacteria in *V. teredinicola* are similar in phylogenetic identity and tissue location to the gill symbionts observed in other bathymodiolin mussels (Duperron et al., 2008b).

The abundance of symbionts observed in the gills of *V. teredinicola* appears to be low in comparison to that observed in some other bathymodiolin mussels, as indicated by the thin and transparent gills, low metagenomic read counts, and sparse detection in FISH images. This sparsity may reflect an inherent characteristic of this host species or a response to environmental conditions, as several *Bathymodiolus* species have been shown to lose or gain symbionts as concentrations of reduced sulfur compounds fluctuate in their environments (Tame et al., 2022). Thus, additional sampling should be performed to determine whether this association is obligate or facultative. Also, as has been shown or inferred for other Bathymodiolinae, *V. teredinicola* may be capable of filter feeding (Guan et al., 2022; Page et al., 1990; von Cosel and Marshall, 2010). Indeed, ingested material is evident within the stomach and digestive glands of sectioned animals (Fig. 5b). In some individuals, green-pigmented material—likely phytoplankton—can be seen in the stomach through the transparent shells (Supplementary Figure SF8). Thus, thioautotrophic symbiosis, heterotrophic feeding, or both may potentially contribute to host nutrition in *V. teredinicola.*

### 3.6. Vadumodiolus teredinicola inhabits shipworm burrows

**Fig. 8.**
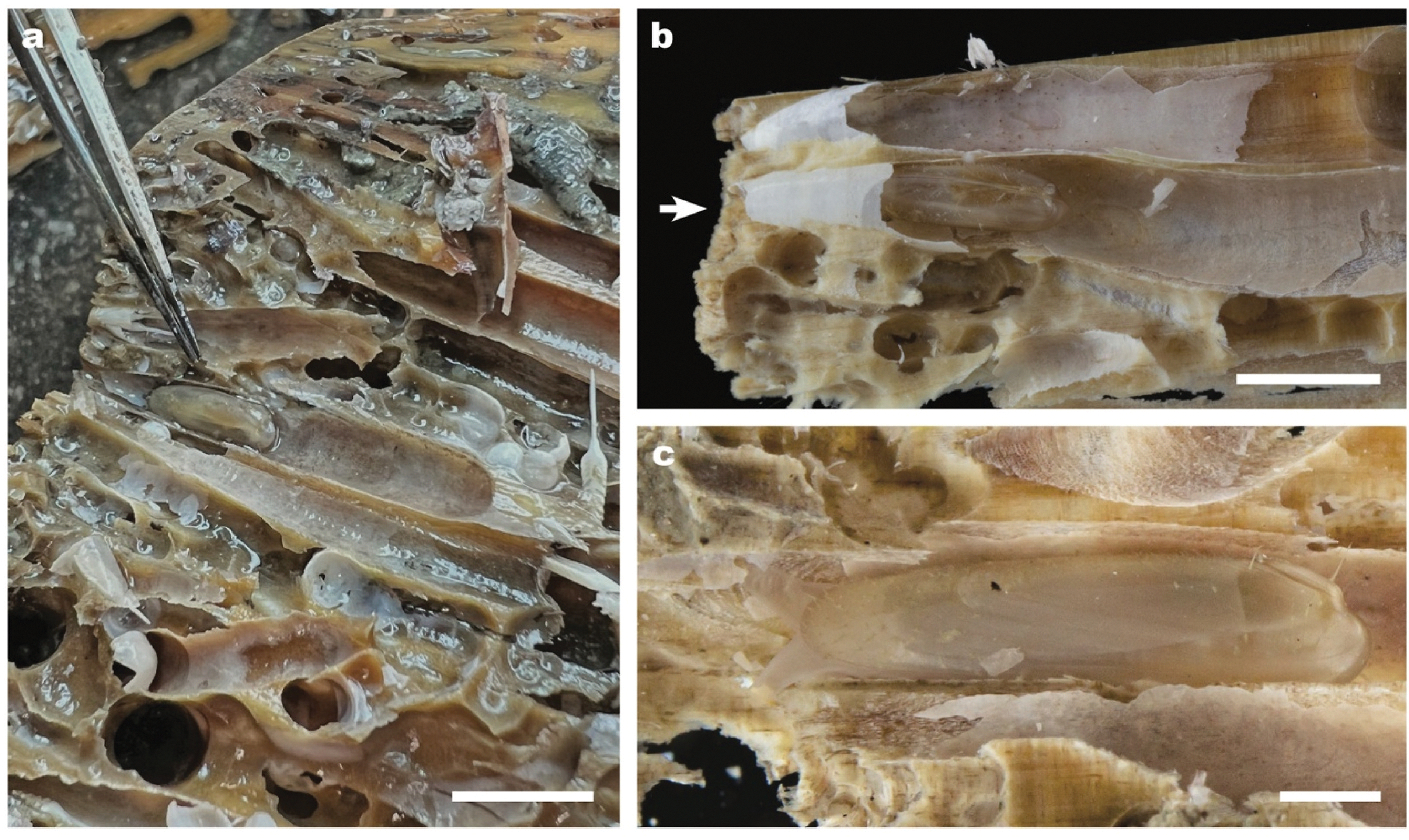
Three specimens of *Vadumodiolus teredinicola* in life position within abandoned shipworm burrows. One side of the surrounding wood and burrow linings have been removed to expose the specimens in the burrows. Arrow shows tip of a calcified burrow entrance. Note that specimens have grown too large to escape from or reorient within the burrows. Scale bars, (a) 10.0 mm, (b) 5.0 mm, (c) 2.0 mm.

All specimens of *V. teredinicola* collected to date were found within unoccupied burrows of the shipworms *Bankia gouldi*, *Nototeredo knoxi,* and *Teredothyra matocotana* in ancient naturally occurring wood and deployed collection panels at the AUF site. Within the burrows, specimens of *V. teredinicola* displayed a highly consistent life position, oriented with their posterior ends facing the conical posterior ends of the shipworm burrows and their siphons extending toward the open burrow entrances (Fig. 8). In most cases, the mussels were too large to exit through the tiny calcareous burrow openings, indicating that they had entered the blind shipworm burrows as larvae or plantigrades and had become captive due to their growth within the burrows. In all but one observed case, where one large and one small specimen shared a single burrow (Supplementary Figure SF9), only one individual mussel was found within a given burrow.

### 3.7. Vadumodiolus teredinicola occurs, grows, and reproduces at shallow depths and warm temperatures

Because specimens must enter the burrows as larvae or plantigrades, and juveniles and adults subsequently become trapped in the shipworm burrows as they grow, the deployment and recovery dates of collection panels can be used to constrain growth and recruitment rates and place bounds on the time to sexual maturity. Within 12 months of burrow entry, specimens grew to the largest sizes observed (~15 mm in length and 4 mm in height) and achieved sexual maturity within the shipworm burrows, as evidenced by microscopic examination of sectioned gonads (Supplementary Figure SF3).

Specimens settled in high abundance within the collection panels. For example, in three roughhewn bald cypress logs (approximately 17 cm diameter × 31 cm length) deployed at the site for 8 months, 16, 25, and 35 specimens were recovered, respectively. Recruitment and survival of *V. teredinicola* was observed in collection panels deployed from December 2019 to June 2020, October 2020 to August 2021, November 2021 to June 2022, and June 2022 to May 2023—a period of 30 months spanning four seasons. Water temperatures during collection panel deployments ranged from 19°C in December 2019 to 28.7-°C in August 2021.

The observed abundance of individuals, including sexually mature adults, and consistent recruitment of *V. teredinicola* in collection panels over multiple seasons provides strong evidence of local growth and reproduction rather than introduction from another locality. The AUF site is located in a relatively shallow region of the Gulf of Mexico, where seafloor depth increases gradually for more than 80 km before dropping off steeply at the edge of the continental shelf (Reese et al., 2018). It has been estimated that larvae of at least one bathymodiolin species, *Gigantidas childressi*, may remain planktonic for up to one year (Arellano and Young, 2009), giving ample time for larvae to drift large distances before settlement. However, the high average recruitment observed in our collection panels (25 ± 9 post-larval individuals per panel over an 8-month deployment, *N* = 3) is inconsistent with dispersal from a distant source. Therefore, it is more likely that *V. teredinicola* grows and reproduces at or near the AUF site at substantially shallower depths and warmer temperatures than those previously demonstrated for bathymodiolin mussels.

### 3.8. Why does V. teredinicola associate with wood?

Wood at the AUF site may serve as shelter and stable substrate for many species, including *V. teredinicola,* that might otherwise be unable to thrive on the constantly shifting and relatively featureless sea floor of the Mississippi-Alabama-Florida sand sheet. The massive quantities of preserved wood found at the submerged forest site also constitute a large reservoir of reduced carbon and energy that can be consumed as food by shipworms and used indirectly by other species. Shipworms convert wood into more available forms, i.e., their own biomass, waste, and reproductive products, which in turn serve as food for a wide range of other organisms. Additionally, the burrows of shipworms and other wood-boring bivalves create protective habitat within the wood that can be used by fish (Hendy et al., 2013) and many invertebrates (Hendy et al., 2014; Wolff, 1979), including bathymodiolin mussels (Dell, 1987). At the AUF site, shipworm burrows were observed to harbor not only *V. teredinicola* but also crabs, shrimps, bivalves, and worms of several phyla.

Wood carbon and energy are also utilized by anaerobic sulfate-reducing bacteria. In consuming organic matter, these bacteria reduce seawater sulfate to sulfides (Bienhold et al., 2013; Ristova et al., 2017). Thus, rotting wood and decaying shipworm carcasses may provide a source of sulfides that the sulfide-oxidizing symbionts of *V. teredinicola* can utilize. Additionally, the shipworm burrows may promote sulfide production by increasing the surface area of the wood for bacterial colonization and by creating confined spaces that become anaerobic, favoring the growth of sulfate-reducing bacteria.

### 3.9. Vadumodiolus teredinicola is well-adapted for life within shipworm burrows

The shells of *V. teredinicola* are exceptionally thin and fragile, making it difficult to handle living specimens without crushing them. These shells would be expected to provide little protection against predators or environmental challenges in more exposed settings. However, within the protective confines of the shipworm burrows, these fragile shells may provide sufficient defense, potentially obviating the need and metabolic cost of producing thicker, more protective shells.

Unlike the shells of many bathymodiolin mussels whose short and narrow anterior ends and tall and wide posterior ends are well adapted to byssal attachment and nestling in shallow crevices, the height and width of the valves of *V. teredinicola* are greatest near the anterior and taper toward the posterior shell margin. The resulting shape is nearly conical in the frontal aspect (Fig. 3d, Supplementary Video SV1). This shape closely approximates the conical shape of the posterior end of the shipworm burrows, allowing individuals to maximize growth within this limited space while maintaining proximity to the conical burrow entrance. The consistent height to length ratio observed among specimens of different sizes suggests that this elongate shape is not the solely the result of the constraints of the burrows as it is shared by smaller specimens, which may be less constrained by burrow dimensions, as well as larger specimens.

The shells of *V. teredinicola* are decorated with densely distributed, flexible periostracal hairs and long, stiff, and very fragile calcified spines that stand erect on the surfaces of the valves. These spines are most prominent on the anterior and posterior ends of the valves (Fig. 3). The spines and periostracal hairs hold the valves away from the walls of the burrow, possibly improving water flow around the animals. The spines may also serve a defensive function, discouraging attack by predators within the narrow confines of shipworm burrows.

Specimens of *V*. *teredinicola* are highly mobile via a long prehensile foot (Supplementary Video SV2), capable of extending at least one body length beyond the anterior edge of the valves. The distal end of the foot forms a shield- or disk-shaped tip that can grasp both rough and smooth surfaces. By repeatedly extending the foot, attaching the tip to surfaces, and contracting the foot, individuals can rapidly crawl over surfaces and can even climb considerable distances up the sides of a glass beaker of seawater. Although a byssal groove is evident near the heel of the foot, byssal threads were not observed, and specimens did not form byssal attachments within shipworm burrows. Thus, the prehensile foot and lack of byssal attachment may facilitate movement along the length of the shipworm burrows, allowing individuals to migrate between the more aerobic entrances and more anaerobic blind ends of shipworm burrows, potentially giving them access to both the sulfides and the oxygen needed to support their thioautotrophic symbionts.

Although many features of *V. teredinicola* suggest specialization for inhabiting shipworm burrows, it has not yet been determined whether this species occurs in other types of crevices for which these features may be equally adaptive. Nonetheless, at minimum, this association represents a non-obligate commensalism, wherein the mussels benefit from the burrows of shipworms, while the shipworms—perhaps having already died prior to entry of the mussels—may be unaffected by the mussels that replace them.

## 4. Conclusions

The results presented here substantially extend the depth and temperature ranges within which bathymodiolin species have been demonstrated to grow and reproduce and show that wood has been involved in the success of Bathymodiolinae in both deep- and shallow-water environments. These results are consistent with the “wooden steps” hypothesis in that they support the proposal that the progenitors of modern deep-sea Bathymodiolinae were likely associated with wood and other organic deposits. However, while modern *V. teredinicola* resides in shallow water, our results do not preclude the possibility that their progenitors followed an evolutionary trajectory from deep to shallow water, in which case wood may have acted as a stepping stone toward shallow marine environments. More comprehensive sampling of shallow environments, especially those associated with submerged wood, will be required to determine whether additional shallow-water bathymodiolin species await discovery.

## Supplementary Material

Online_supplement

Vt_motility.compressed

microCT_scan.compressed

Vt_motility

Vt_microCT_scan

## Data Availability

All data used may be found herein or are posted to GenBank (accession numbers in main text and supplement) or Dryad: https://datadryad.org/stash/share/HaEl2zZB1mSEZy0lMge2zsUe7KGH2Eb24L5_7HGypIo.

## Appendix A. Supplementary data

Supplementary data to this article can be found online at https://doi.org/10.1016/j.dsr.2023.104220.
