# Supplementary material for: Wooden steps to shallow depths: A new bathymodiolin mussel, Vadumodiolus teredinicola, inhabits shipworm burrows in an ancient submarine forest
Source: Deep Sea Res 1 Oceanogr Res Pap. Author manuscript; Available in PMC 2026 Jan 14. (PMC12798760; doi:10.1016/j.dsr.2023.104220)
Supplement: Online_supplement [file NIHMS2051314-supplement-Online_supplement.pdf]

# Online Supplement

**Wooden steps to shallow depths: a new bathymodiolin mussel, *Vadumodiolus teredinicola*, inhabits shipworm burrows in an ancient submarine forest**

Marvin A. Altamia[1], Hannah J. Appiah-Madson[1], Rosalia Falco Poulin[1], Bruno Huettel[2], Maxim Rubin-Blum[3], Nicole Dubilier[4], Harald R. Gruber-Vodicka[4], Nikolaus Leisch[4], and Daniel L. Distel[1*]

Affiliations

[1] Ocean Genome Legacy Center, Department of Marine and Environmental Science, Northeastern University, Nahant, MA, USA

[2]Max Planck Genome Centre Cologne, Max Planck Institute for Plant Breeding Research, Cologne, Germany

[3]Israel Oceanographic and Limnological Research Institute– IOLR, Haifa, Israel

[4] Max Planck Institute for Marine Microbiology, Bremen, Germany

*Corresponding author d.distel@northeastern.edu

Keywords: Bathymodiolinae, Bivalve evolution, Chemoautotrophic symbiosis, Mytilidae, Teredinidae, Thioautotrophic symbionts

# Supplementary Figures

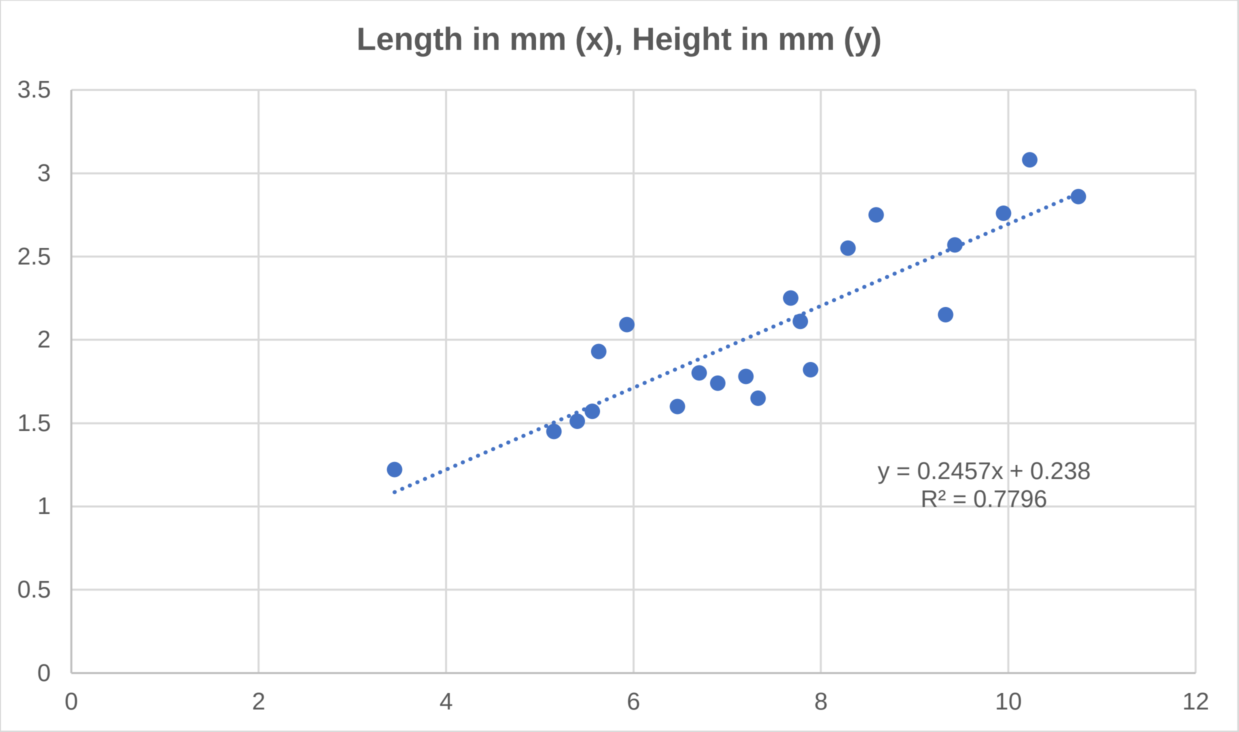


**Supplementary Figure SF1. Length and height measurements for the valves of the *Vadumodiolus teredinicola* type series.** Scatter plot showing length (x axis) and height (y axis) of valves in mm for 21 specimens of *V. teredinicola* collected from a bald cypress log exposed for 8 months at the Alabama undersea forest site. Numerical values and associated specimen data are shown in Supplementary Table ST2.

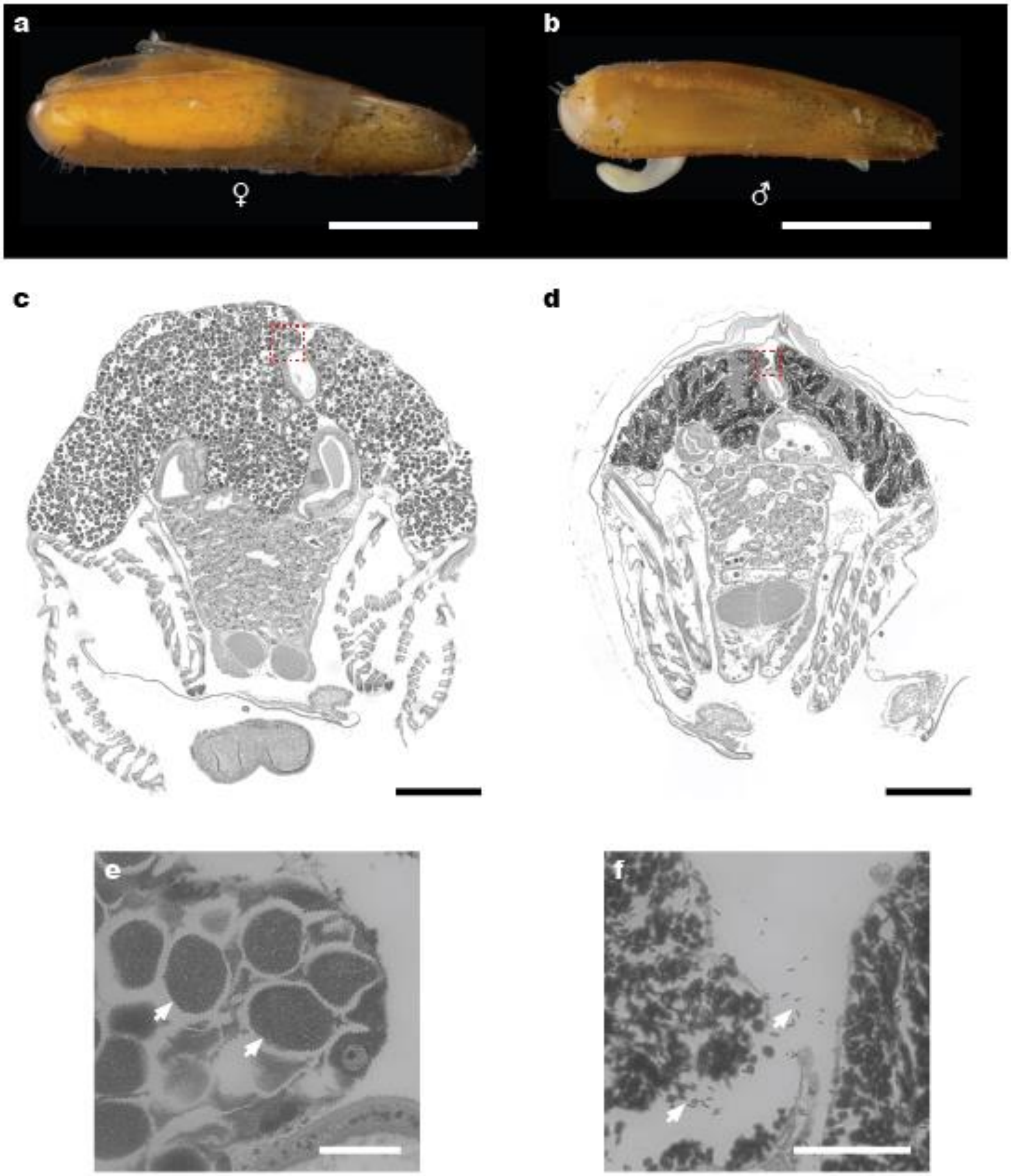


**Supplementary Figure SF2. Female and male specimens of *Vadumodiolus teredinicola.*** (a) female specimen, (b) male specimen, (c-d) hematoxylin- and eosin-stained transverse sections through the visceral mass and gills of a (c) female and (d) male specimen. (e) detail of the dashed boxes in (c), (f) detail of the dashed box in (d). Arrows in (e) and (f) indicate mature oocytes and sperm cells, respectively. Scale bars, (a, b) 5.0 mm, (c, d) 500 µm, (e, f) 40 µm.

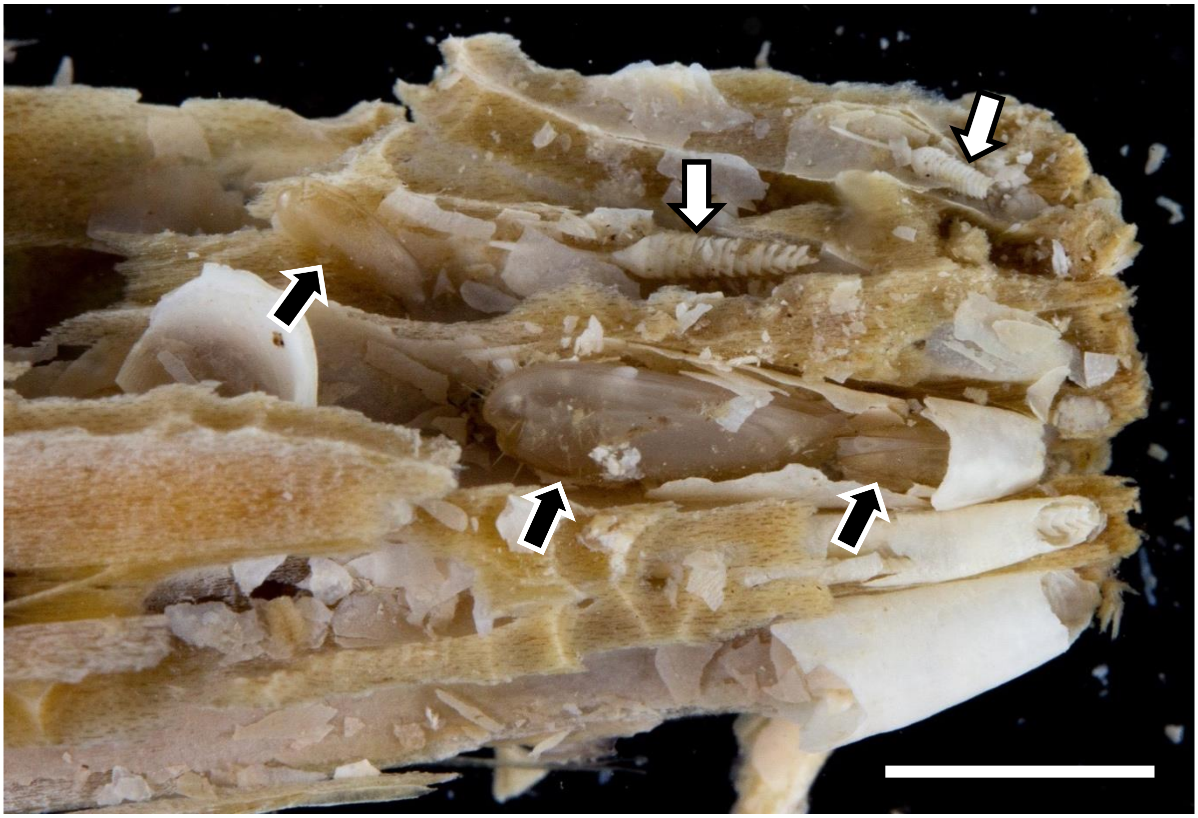

**Supplementary Figure SF3**: **Specimens of *Vadumodiolus teredinicola* in abandoned shipworm burrows.** The left sides of the burrow walls have been removed to show the specimens in life position within the burrows. Black arrows indicate *V. teredinicola* specimens. White arrows indicate the remaining pallets *Bankia gouldi,* the shipworm species that created and inhabited the burrows previously. Scale bar = 5.0 mm.

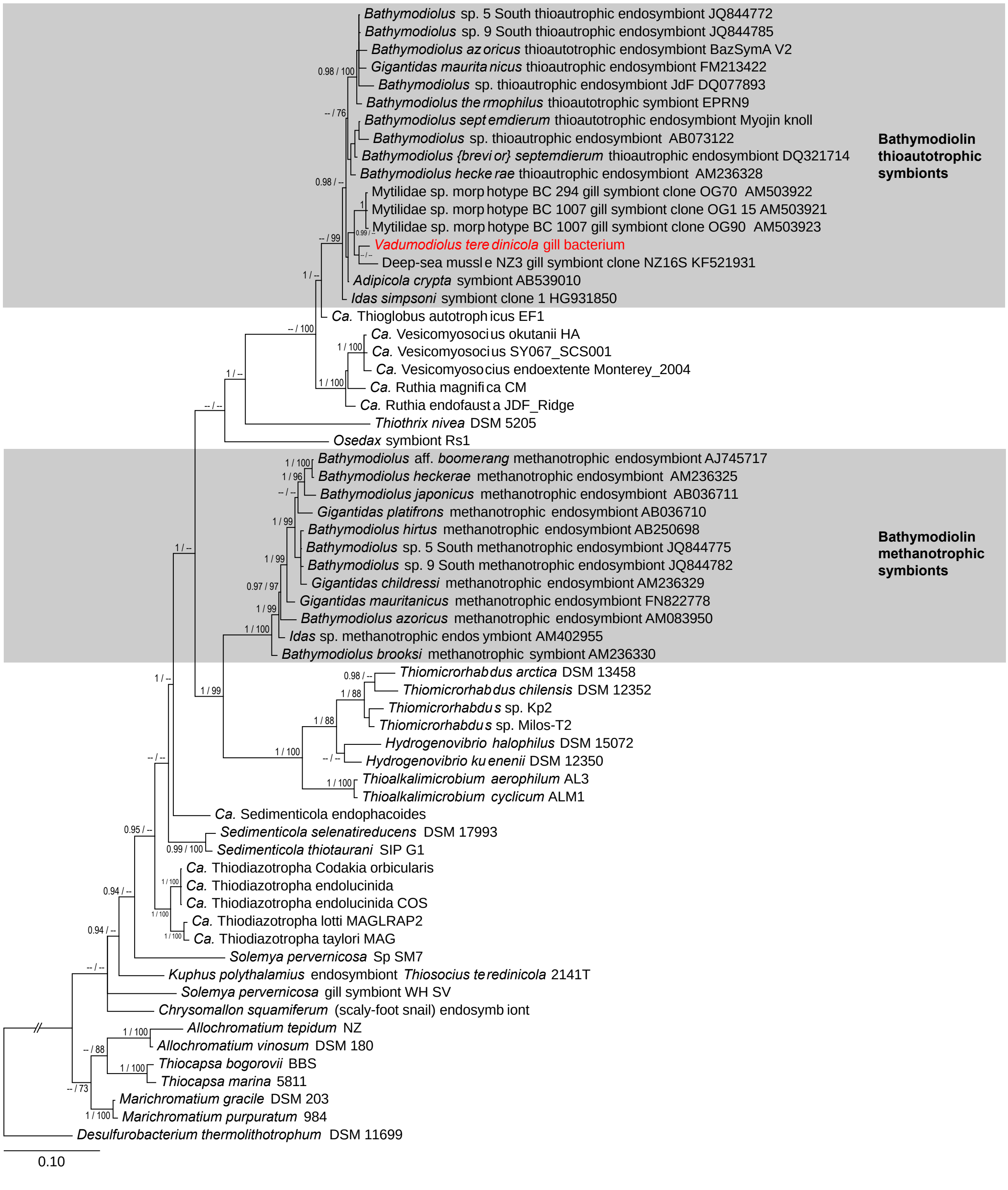
Bathymodiolus sp. 5 South thioautrophic endosymbiont JQ844772
Bathymodiolus sp. 9 South thioautrophic endosymbiont JQ844785
Bathymodiolus azoricus thioautotrophic endosymbiont BazSymA V2
Gigantidas mauritanicus thioautrophic endosymbiont FM213422
Bathymodiolus sp. thioautrophic endosymbiont JdF DQ077893
Bathymodiolus thermophilus thioautotrophic symbiont EPRN9
Bathymodiolus septemdierum thioautotrophic endosymbiont Myojin knoll
Bathymodiolus sp. thioautrophic endosymbiont AB073122
Bathymodiolus {brevior} septemdierum thioautrophic endosymbiont DQ321714
Bathymodiolus heckerae thioautrophic endosymbiont AM236328
Mytilidae sp. morphotype BC 294 gill symbiont clone OG70 AM503922
Mytilidae sp. morphotype BC 1007 gill symbiont clone OG1 15 AM503921
Mytilidae sp. morphotype BC 1007 gill symbiont clone OG90 AM503923
Vadumodiolus teredinicola gill bacterium
Deep-sea mussle NZ3 gill symbiont clone NZ16S KF521931
Adipicola crypta symbiont AB539010
Idas simpsoni symbiont clone 1 HG931850
Ca. Thioglobus autotrophicus EF1
Ca. Vesicomyosocius okutanii HA
Ca. Vesicomyosocius SY067_SCS001
Ca. Vesicomyosocius endoextente Monterey_2004
Ca. Ruthia magnifica CM
Ca. Ruthia endofausta JDF_Ridge
Thiothrix nivea DSM 5205
Osedax symbiont Rs1
Bathymodiolus aff. boomerang methanotrophic endosymbiont AJ745717
Bathymodiolus heckerae methanotrophic endosymbiont AM236325
Bathymodiolus japonicus methanotrophic endosymbiont AB036711
Gigantidas platifrons methanotrophic endosymbiont AB036710
Bathymodiolus hirtus methanotrophic endosymbiont AB250698
Bathymodiolus sp. 5 South methanotrophic endosymbiont JQ844775
Bathymodiolus sp. 9 South methanotrophic endosymbiont JQ844782
Gigantidas childressi methanotrophic endosymbiont AM236329
Gigantidas mauritanicus methanotrophic endosymbiont FN822778
Bathymodiolus azoricus methanotrophic endosymbiont AM083950
Idas sp. methanotrophic endosymbiont AM402955
Bathymodiolus brooksi methanotrophic symbiont AM236330
Thiomicrorhabdus arctica DSM 13458
Thiomicrorhabdus chilensis DSM 12352
Thiomicrorhabdus sp. Kp2
Thiomicrorhabdus sp. Milos-T2
Hydrogenovibrio halophilus DSM 15072
Hydrogenovibrio kuenenii DSM 12350
Thioalkalimicrobium aerophilum AL3
Thioalkalimicrobium cyclicum ALM1
Ca. Sedimenticola endophacoides
Sedimenticola selenatireducens DSM 17993
Sedimenticola thiotaurani SIP G1
Ca. Thiodiazotropha Codakia orbicularis
Ca. Thiodiazotropha endolucinida
Ca. Thiodiazotropha endolucinida COS
Ca. Thiodiazotropha lotti MAGLRAP2
Ca. Thiodiazotropha taylori MAG
Solemya pervernicosa Sp SM7
Kuphus polythalamius endosymbiont Thiosocius teredinicola 2141T
Solemya pervernicosa gill symbiont WH SV
Chrysomallon squamiferum (scaly-foot snail) endosymbiont
Allochromatium tepidum NZ
Allochromatium vinosum DSM 180
Thiocapsa bogorovii BBS
Thiocapsa marina 5811
Marichromatium gracile DSM 203
Marichromatium purpuratum 984
Desulfurobacterium thermolithotrophum DSM 11699
Bathymodiolin thioautotrophic symbionts
Bathymodiolin methanotrophic symbionts
0.10

**Supplementary Figure SF4: Phylogram depicting the inferred relationships among *Vadumodolius teredinicola* gill bacterium and related symbionts based on 16S rRNA sequences.** Sequences were aligned using MAFFT auto algorithm with the scoring matrix 200PAM / k-2, gap open penalty of 1.53 and offset value of 0.123. Aligned sequences were trimmed to 1,314 bp. The phylogram presented is a Bayesian tree employing GTR+I+Γ as the substitution model in MrBayes version 3.2.6. A maximum-likelihood tree was also constructed using IQ-Tree2 with GTR+I+Γ as the substitution model. Posterior probability values greater than 0.90 are displayed to the left of the slash at associated nodes. To the right of the slash are bootstrap proportions greater than 70 percent of 1000 replicates for nodes also supported by the maximum likelihood analysis (iQ-Tree v2.2). Scale bar = 0.1 substitutions per 100 base pairs. NCBI accession numbers for symbionts known only by their 16S rRNA sequences are indicated at the end of taxon name.

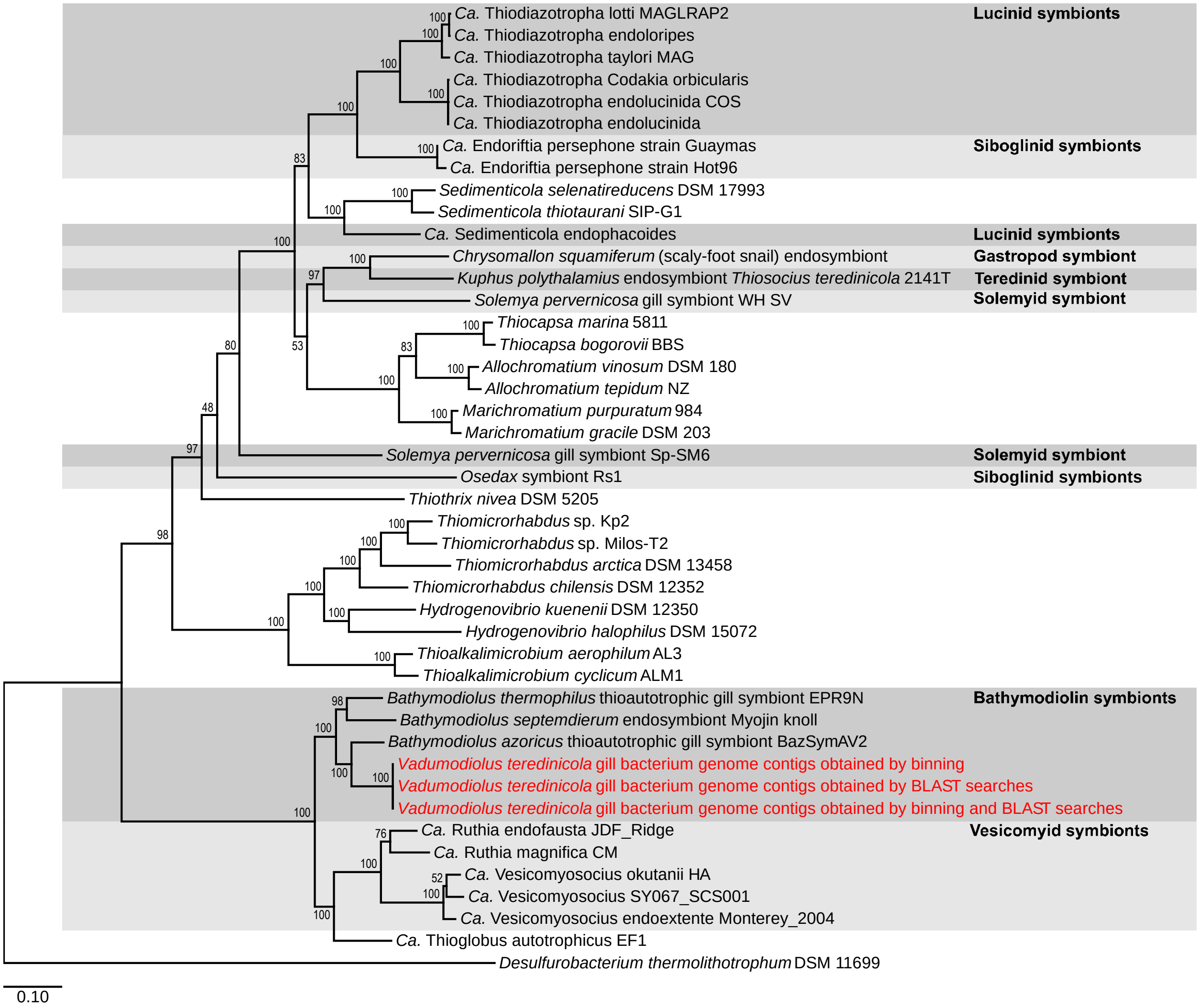


**Supplementary Figure SF5: Phylogram depicting the inferred relationships among three *Vadumodolius teredinicola* gill bacterium metagenome assembled genome assemblies and related symbionts based conserved protein markers.** The Genome Taxonomy Database Tool Kit was used to identify, extract, and align the protein markers in the genome assemblies. The maximum likelihood tree was constructed using RAxML. Bootstrap proportions greater than 70 percent of 1,000 replicates are indicated for each node. The scale bar represents the substitution rate per site. Note that the *V. teredinicola* genome assemblies obtained by Metabat2 binning, BLAST searches, and a combination of both, are indistinguishable in this analysis.

**a**

| ANI Matrix (above) / AAI Matrix (below) | *V. teredinicola* | *B. azoricus* | *B. septemdierum* | *B. thermophilus* | *Ca.* T. autotrophica | *Ca.* R. magnifica | *Ca.* V. okutanii | *Ca.* V. endoextente |
|---|---|---|---|---|---|---|---|---|
| *V. teredinicola* gill bacterium | | 81 | 77 | 79 | 77 | 77 | 77 | 77 |
| *B. azoricus* symbiont | 82 | | 78 | 80 | 77 | 77 | 77 | 77 |
| *B. septemdierum* symbiont | 77 | 77 | | 79 | 77 | 76 | 77 | 77 |
| *B. thermophilus* symbiont | 78 | 77 | 77 | | 77 | 76 | 77 | 77 |
| *Ca. Thioglobus autotrophica* | 73 | 71 | 72 | 71 | | 77 | 76 | 76 |
| *Ca. Ruthia magnifica* | 72 | 72 | 72 | 72 | 74 | | 83 | 83 |
| *Ca. Vesicomyosocius okutanii* | 70 | 71 | 70 | 71 | 73 | 83 | | 94 |
| *Ca. Vesicomyosocius endoextente* | 70 | 71 | 70 | 71 | 72 | 82 | 95 | |

**b**

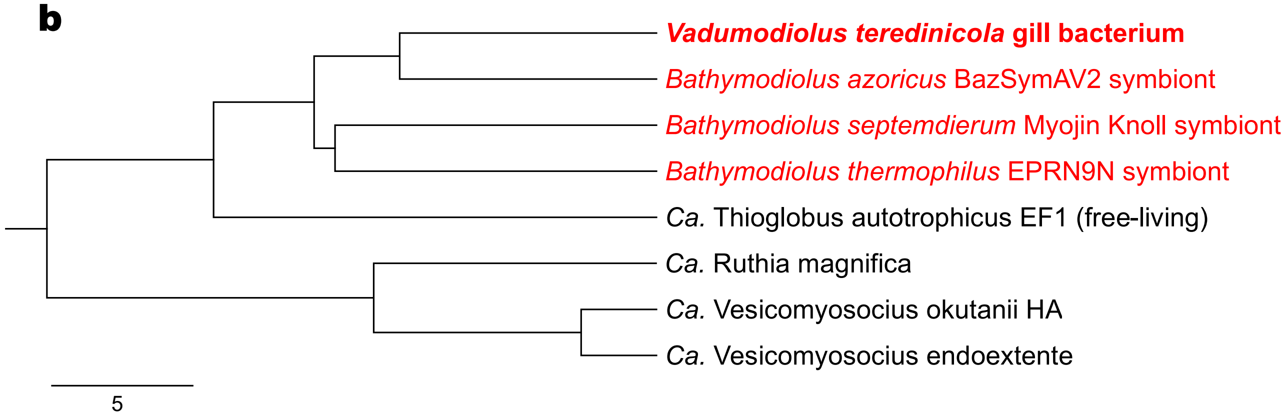


**c**

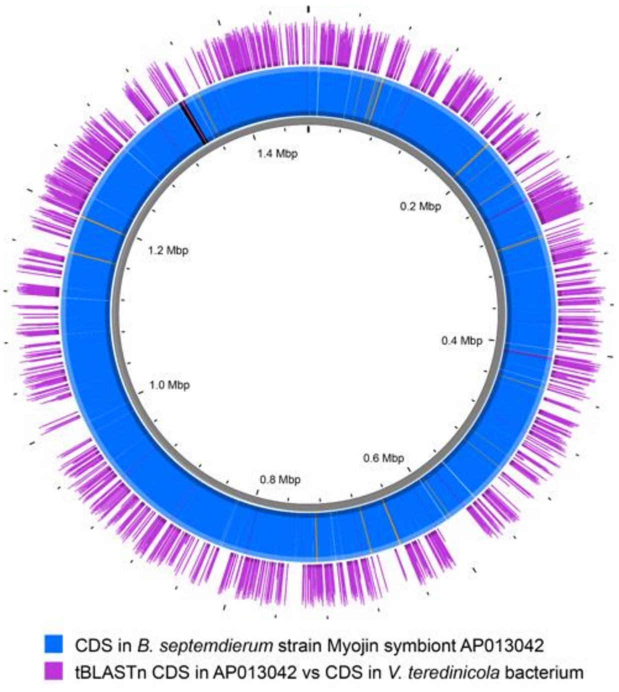

**Supplementary Figure SF6: Comparison of the *Vadumodiolus teredinicola* gill bacterium metagenome-assembled genome (MAG) with other bathymodiolin symbiont MAGs.** (a) Heat map showing comparisons of genomic average nucleotide identity (ANI; above) and average amino identity acid (AAI; below) among bathymodiolin symbionts and related bacteria. Color scale is from green (lowest) to red (highest). (b) ANI-distance clustering based on the Ward method. Note that the *V. teredinicola* gill bacterium clusters with other bathymodiolin thioautotrophic symbionts. (c) Alignment of coding sequences identified in the *V. teredinicola* gill bacterium MAG (magenta) with the MAG of *Bathymodiolus septemdierum* Myojin Knoll symbiont closed-circular genome (blue).

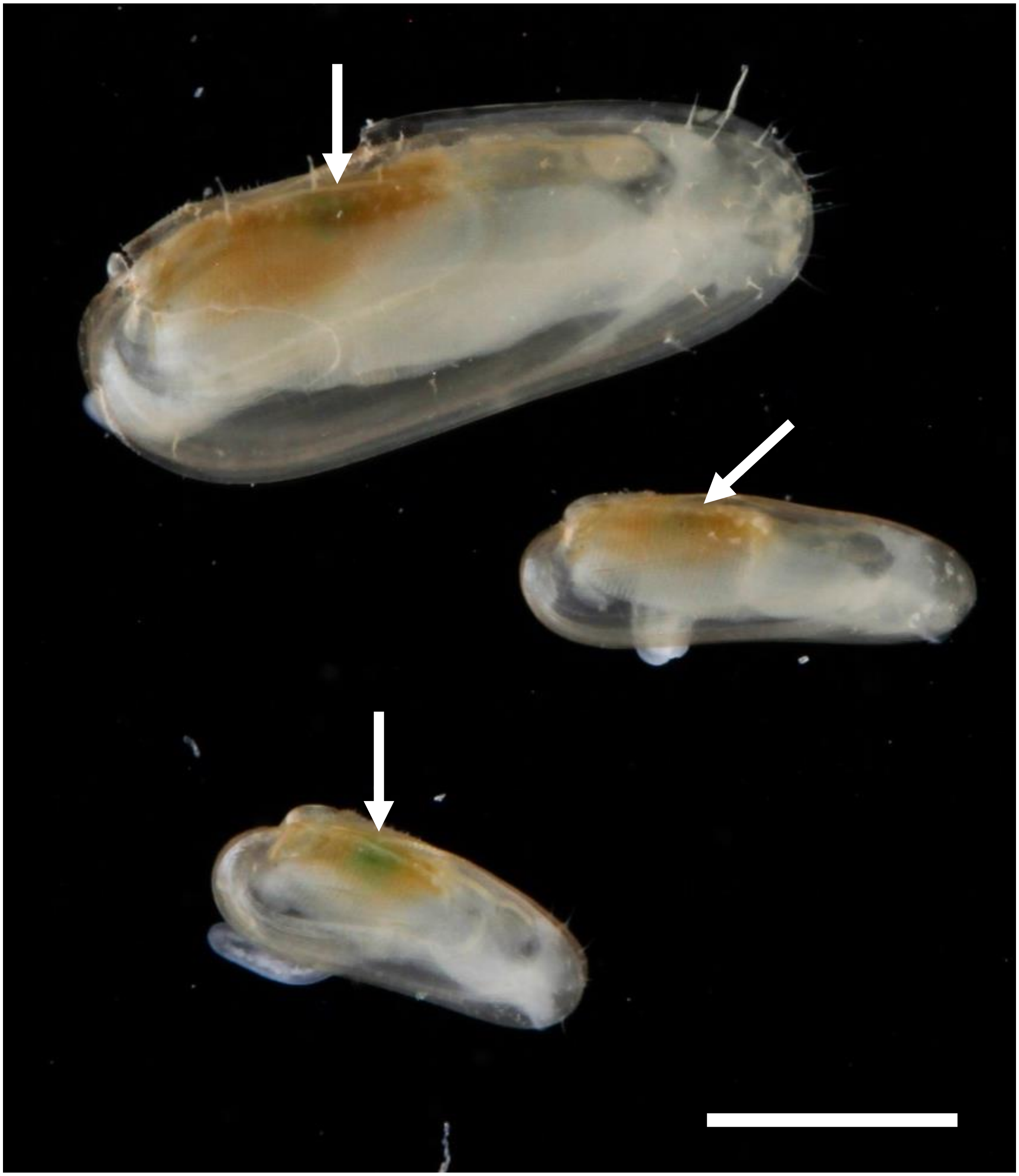

**Supplementary Figure SF7. Specimens of *Vadumodiolus teredinicola* showing green pigmented material within the stomach.** Scale bar = 2.0 mm.

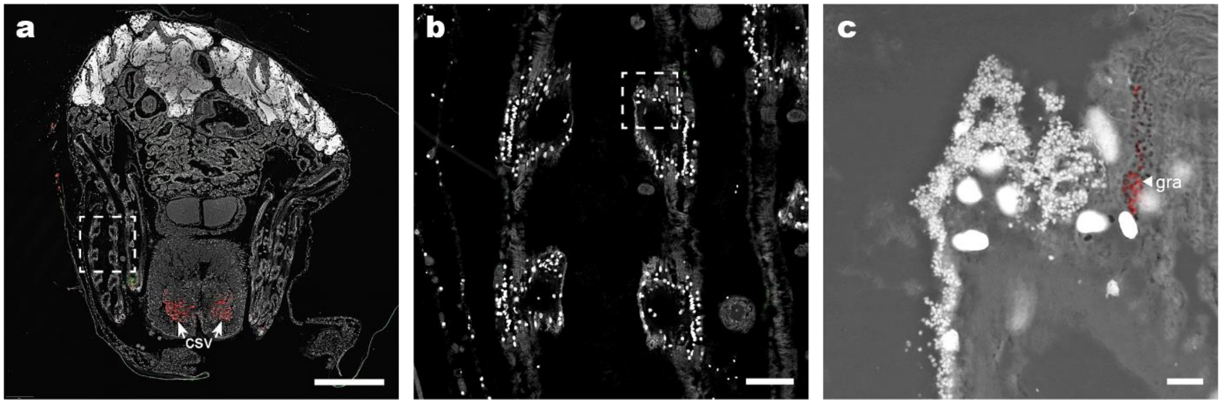


**Supplementary Figure SF8: Confocal laser scanning microscopy overview of the symbionts of *Vadumodiolus teredinicola* using antisense probes.** Dual-probe fluorescence *in situ* hybridization (FISH) using 16S rRNA antisense probes Cy5-EUB338NON (red) and Cy3-EUB338NON (green). DNA is stained with NucBlue Nuclear Stain (white). (a) transverse section through the gill and visceral mass of a mature male specimen of *V. teredinicola*, (b) detail of the boxed region in (a), (c) detail of the boxed region in (b). Note the absence of antisense probe signals in the gill bacteria (boxed regions). Autofluorescence is observed in the cuticle secretory vesicles (csv) in the foot and granulocytes (gra) in the gills.

## Supplementary Videos

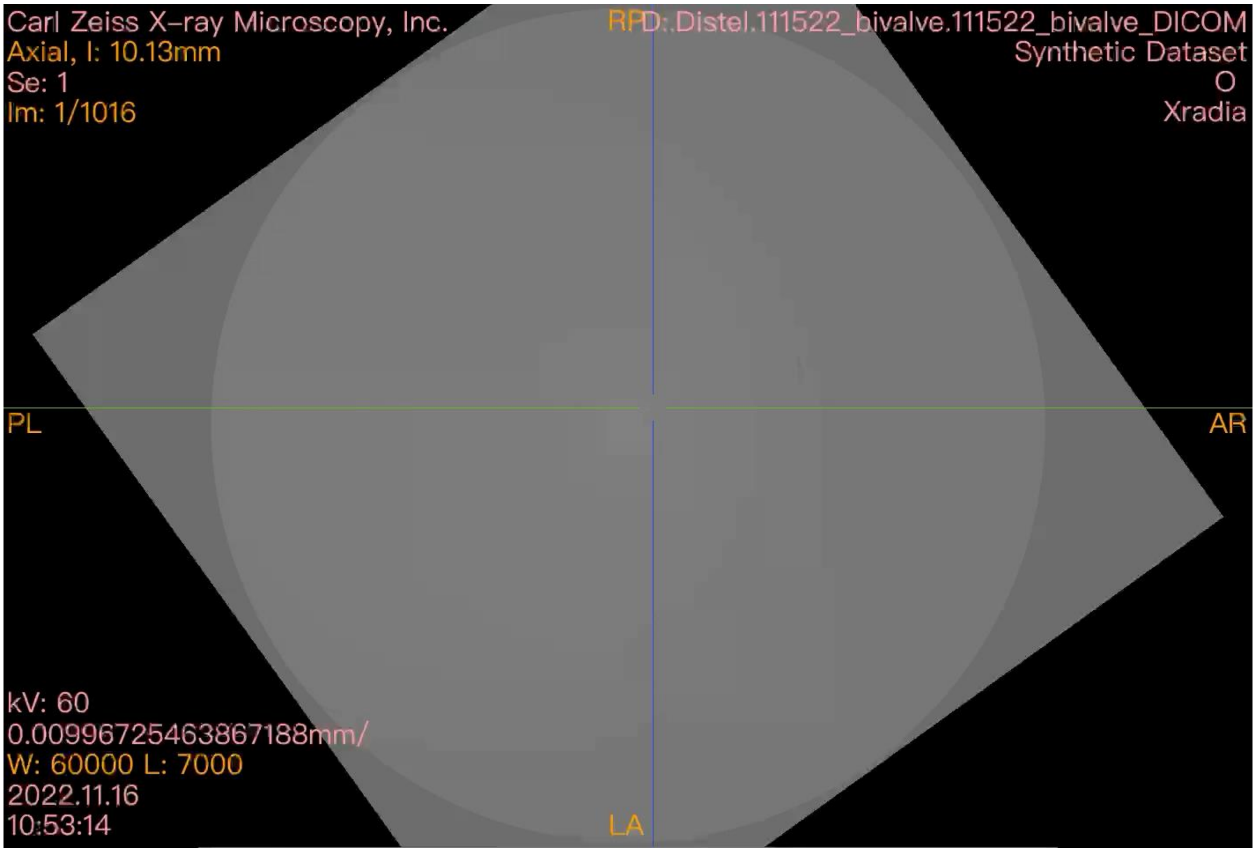


**Supplementary Video SV1. Animation demonstrating the anatomy of *Vadumodiolus teredinicola* as visualized by microcomputed tomography.** Animation shows 830 virtual transverse sections through a specimen of *V. teredinicola.* Sections are spaced at intervals of approximately 9.6 µm and are ordered from posterior to anterior. The specimen is approximately 8.59 mm in length and appears in frames 86-916. Video may be found at https://datadryad.org/stash/share/HaEl2zZB1mSEZy0lMge2zsUe7KGH2Eb24L5_7HGypIo.

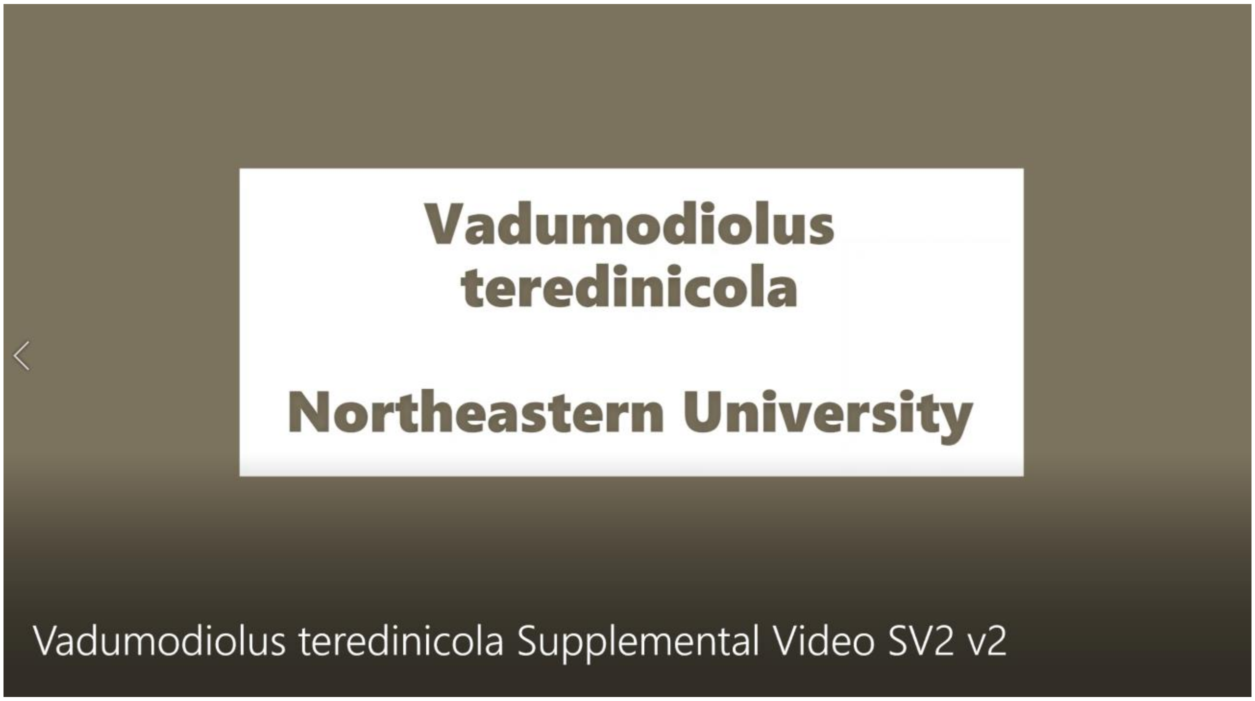


**Supplementary Video SV2. Motility of *Vadumodiolus teredinicola*.** Specimens of *V. teredinicola* are highly mobile via a long prehensile foot, capable of extending at least one body length beyond the anterior edge of the valves. The distal end of the foot forms a muscular disk- or shield-shaped surface that can grasp both rough and smooth surfaces. By repeatedly extending the foot, attaching the tip to surfaces, and contracting the foot, individuals can rapidly crawl over surfaces. Video may be found at https://datadryad.org/stash/share/HaEl2zZB1mSEZy0lMge2zsUe7KGH2Eb24L5_7HGypIo.

## Metagenome assembly details

To search for evidence of bacterial symbionts in the tissues of *Vadumodiolus teredinicola*, we sequenced the gill metagenomes of three specimens, assembled contigs and sorted contigs of predicted bacterial origin into bins reflecting individual predicted bacterial MAGs using SPADES and metaBAT2.

Reads of bacterial origin were sparsely represented in the three individual gill metagenome datasets. However, by combining reads from all three gill metagenome sequences during SPADES assembly, a single, though highly fragmented, bacterial MAG was assembled. MetaBAT2 uses tetranucleotide composition information to perform binning. MetaBAT2 takes a metagenome assembly and the reads that produced the assembly and organizes the contigs into putative genomes, called "bins". Because metaBAT2 only uses contigs longer than 1.5 kbp during binning, we performed BLASTn searches using the 16S rRNA genes, 23S rRNA genes, and coding sequences (CDS) of *B. thermophilus* thioautotrophic gill symbiont strain EPR9N to recruit un-binned short bacterial contigs in the assembled metagenome. A maximum likelihood phylogenetic analysis (RAxML) based on a set of 120 conserved bacterial genes identified and aligned using GTDB-tk (Chaumeil, Mussig et al. 2019) showed that the metaBAT2 bin and the contigs identified with BLAST searches are nearly identical and that they form a well-supported clade (100% bootstrap support, 1,000 replicates) with symbiont genomes determined for *Bathymodiolus azoricus, B. septemdierum* and *B. thermophilus* (Supplementary Table ST2-4, Supplementary Figure SF3). Because the metaBAT2 binned bacterial contigs and concatenated contigs obtained using BLAST searches are phylogenetically indistinguishable, it is reasonable to assume that they are represent the

same bacterial genome present in the gills, and thus can be combined to create a more complete assembly. The final assembly is composed of 611 contigs with a total size of 1,060,823bp and a %GC content of 38.4%. The *V. teredinicola* gill bacterial final draft genome assembly is highly fragmented with an N50 of just 2.3 kbp, nevertheless it is estimated to be 87.88% complete with an acceptable level of contamination of just 2.82% (Supplementary Table ST2). The draft *V. teredinicola* gill bacterium MAG is deposited online at NCBI as WGS Assembly under accession number JASISB000000000 (BioSample SAMN35084530, BioProject PRJNA964767).

In most instances, rRNA genes cannot be recovered in the metagenomes if there is a complex community present. However, in *V. teredinicola*, both near full-length 16S and 23S rRNA genes can be recovered indicating that the gill microbial community is composed of a single symbiont ribotype. This is similar to what has been observed in *B. septemdierum* and *B. thermophilus* wherein only 1 type of thioautotrophic symbiont is present. We identified homologs of sulfide-quinone reductase (*sqr*), adenylylsulfate reductase subunit alpha (*aprA*), and small and large-subunits of ribulose-1,5-bisphosphate carboxylase/oxygenase (RuBisCO) (*cbbS* and *cbbL*) in the genome assembly, signifying that the *V. teredinicola* gill bacterium is a thioautotroph, like many bathymodiolin symbionts (Supplementary Table ST3).

## Supplementary Tables

**Supplementary Table ST1: GenBank accession records for taxa and genes used in this study.**

| Subfamily | Genus | Species | 18S rRNA accession | 28S rRNA accession | 16S rRNA accession | COI accession |
|---|---|---|---|---|---|---|
| Bathymodiolinae | *Vadumodiolus* | *teredinicola* | OQ919029 | OQ919032 | OQ920280 | OQ915218 |
| Bathymodiolinae | *Idas* | sp. PD Med 12 | OQ919030 | OQ919033 | OQ920281 | OQ915219 |
| Bathymodiolinae | *Idas* | sp. PD Med 12 | OQ919031 | OQ919034 | OQ920282 | OQ915220 |
| Bathymodiolinae | *Idas* | *macdonaldi* | AF221647 | AY781145 | HF545092 | AY649804 |
| Bathymodiolinae | *Idas* | *modiolaeformis* | KF611730 | FJ159555 | KF611772 | FJ158585 |
| Bathymodiolinae | *Adipicola* | *iwaotakii* A" | KF611729 | EU683288 | KF611770 | EU702333 |
| Bathymodiolinae | *Idas* | ESU N | KF611728 | GU065843 | KF611769 | FJ937205 |
| Bathymodiolinae | *Idas* | sp. D | KF611724 | EU683275 | KF611765 | EU702357 |
| Bathymodiolinae | *Idas* | sp. C | KF611725 | EU683260 | KF611766 | EU702376 |
| Bathymodiolinae | *Idas* | ESU M | KF611726 | GU065845 | KF611767 | FJ937202 |
| Bathymodiolinae | *Idas* | ESU L | KF611727 | GU065767 | KF611768 | FJ937193 |
| Bathymodiolinae | *Idas* | SAL1 | DQ340794 | DQ863944 | KF611761 | DQ340775 |
| Bathymodiolinae | *Idas* | ESU P | KF611721 | GU065846 | KF611762 | FJ937222 |
| Bathymodiolinae | *Idas* | *washingtonius* | AF221645 | AY781146 | HF545073 | AY275546 |
| Bathymodiolinae | *Idas* | ESU O | KF611722 | GU065763 | KF611763 | FJ937211 |
| Bathymodiolinae | *Idas* | ESU K | KF611723 | GU065868 | KF611764 | FJ937192 |
| Bathymodiolinae | *Idas* | *argenteus* LD | LM992894 | LM992896 | Not available | LM992892 |
| Bathymodiolinae | *Idas* | *argenteus* MAR | LM992893 | LM992895 | Not available | LM992891 |
| Bathymodiolinae | *Terua* | *arcuatilis* | KF611719 | GU065879 | KF611756 | FJ937033 |
| Bathymodiolinae | *Terua* | ESU T | KF611720 | GU065804 | KF611757 | FJ937283 |
| Bathymodiolinae | *Tamu* | *fisheri* | AF221642 | AY781132 | HF545065 | AY649803 |
| Bathymodiolinae | *"Lignomodiolus"* | SAL4 | DQ340796 | DQ863947 | KF611741 | DQ340776 |
| Bathymodiolinae | *"Lignomodiolus"* | ESU R | KF611708 | GU065877 | KF611742 | FJ937239 |
| Bathymodiolinae | *"Lignomodiolus"* | ESU S" | KF611707 | GU065829 | KF611740 | FJ937258 |
| Bathymodiolinae | *"Lignomodiolus"* | ESU Q | KF611709 | GU065875 | KF611743 | FJ937230 |
| Bathymodiolinae | *"Lignomodiolus"* | ESU G | KF611710 | GU065778 | KF611744 | FJ937161 |
| Bathymodiolinae | *Nypamodiolus* | ESU I | KF611716 | GU065774 | KF611754 | FJ937188 |
| Bathymodiolinae | *Nypamodiolus* | *japonicus* (ESU H) | KF611717 | GU065856 | KF611755 | FJ937073 |
| Bathymodiolinae | *Nypamodiolus* | ESU J | KF611715 | GU065842 | KF611753 | FJ937189 |
| Bathymodiolinae | *Nypamodiolus* | *samadiae* | ON508870 | ON508873 | ON508854 | ON491594 |

| | | | | | | |
|---|---|---|---|---|---|---|
| Bathymodiolinae | *Nypamodiolus* | *longissimus* | DQ340798 | DQ863945 | KF611752 | DQ340773 |
| Bathymodiolinae | *Nypamodiolus* | *simpsoni* | KF611731 | KF611700 | KF611773 | KF611695 |
| Bathymodiolinae | *Bathymodiolus* | *puteoserpentis* | AF221640 | AY781151 | HF545053 | AY649796 |
| Bathymodiolinae | *Bathymodiolus* | *azoricus* | AY649822 | AY781148 | KF611758 | AY649795 |
| Bathymodiolinae | *Bathymodiolus* | *heckerae* BR | AY649830 | AY781139 | Not available | AY649793 |
| Bathymodiolinae | *Bathymodiolus* | *brooksi* AC | AY649826 | AY781136 | NC_059706 | AY649797 |
| Bathymodiolinae | *Bathymodiolus* | {*brevior*} *septemdierum* | AY649824 | AY781150 | KX713195 | AY649799 |
| Bathymodiolinae | *Bathymodiolus* | {*marisindicus*} *septemdierum* | AY649818 | AY781147 | NC_059708 | AY275543 |
| Bathymodiolinae | *Bathymodiolus* | *thermophilus* A | AF221638 | AY781141 | HF545052 | AF456285 |
| Bathymodiolinae | *Gigantidas* | *mauritanicus* | KF611712 | FJ890504 | KF611747 | FJ890502 |
| Bathymodiolinae | *Gigantidas* | *childressi* | AF221641 | AY781137 | DQ177885 | AY649800 |
| Bathymodiolinae | *Gigantidas* | *tangaroa* | AY649820 | AY781134 | KF611748 | AY608439 |
| Bathymodiolinae | *Gigantidas* | *taiwanensis* | KF611711 | GU966641 | KF611746 | GU966638 |
| Bathymodiolinae | *Gigantidas* | *gladius* | AY649821 | AY781149 | HF545085 | AY649802 |
| Bathymodiolinae | *Gigantidas* | sp. 2 Broken Bay | KF611713 | KF611697 | KF611749 | KF611692 |
| Bathymodiolinae | *Bathymodiolus "Nipponiomodiolus"* | *manusensis* | KF611718 | GU966642 | KY270856 | GU966637 |
| Bathymodiolinae | *Bathymodiolus "Nipponiomodiolus"* | *aduloides* | Not available | HF545036 | HF545060 | HF545118 |
| Bathymodiolinae | *Adipicola* | *crypta* B" | KF611714 | EU683298 | KF611750 | EU702319 |
| Bathymodiolinae | *Vulcanidas* | ESU E | KF611704 | GU065791 | KF611736 | FJ937079 |
| Bathymodiolinae | *Vulcanidas* | ESU F | KF611705 | GU065809 | KF611737 | FJ937127 |
| Bathymodiolinae | *Vulcanidas* | SAL3 | DQ340800 | DQ863946 | KF611738 | DQ340772 |
| Bathymodiolinae | *Vulcanidas* | *insolatus* | KF611706 | FJ767937 | KF611739 | FJ767936 |
| Bathymodiolinae | *Benthomodiolus* | *lignocola* | AF221648 | AY781131 | KF611733 | AY275545 |
| Bathymodiolinae | *Benthomodiolus* | sp. South Atlantic | KF611703 | KF611698 | KF611733 | KF611691 |
| Bathymodiolinae | *Benthomodiolus* | sp. Juan de Fuca | KF611702 | KF611699 | KF611734 | KF611694 |
| Modiolinae | *Modiolus* | *auriculatus* | KY081336 | KY081360 | Not available | KY081298 |
| Modiolinae | *Modiolus* | *rumphii* | KC429330 | KC429423 | KC429248 | KC429094 |
| Modiolinae | *Modiolus* | *modiolus* | KF611701 | EF526455 | KF611732 | FJ890501 |
| Lithophaginae | *Leiosolenus* | *curtus* | AB201235 | AB103123 | JQ267791 | AB076944 |
| Lithophaginae | *Leiosolenus* | *mucronata* | KY081333 | KY081357 | KY081317 | KY081297 |
| Lithophaginae | *Leiosolenus* | *lima* | KY081331 | KY081354 | KY081316 | KY081294 |
| Mytilinae | *Mytilus* | *edulis* | KC429331 | KC429424 | KC429249 | KC429095 |

**Supplementary Table ST2: Morphometric measurements of the valves of the *Vadumodiolus teredinicola* type series.**

| Specimen Code | OGL Specimen ID | Length (mm) | Height (mm) | Length/Height ratio | Designation | Figure |
|---|---|---|---|---|---|---|
| 6317L-A | S36415 | 10.75 | 2.86 | 3.76 | paratype | 4d |
| 6317L-B | S36416 | 10.23 | 3.08 | 3.32 | holotype | 4a |
| 6317L-C | S36417 | 8.59 | 2.75 | 3.12 | paratype | 4c, 5 |
| 6317L-D | S36418 | 9.43 | 2.57 | 3.67 | paratype | 4b |
| 6317L-E | S36419 | 9.95 | 2.76 | 3.61 | paratype | |
| 6317L-F | S36420 | 9.33 | 2.15 | 4.34 | paratype | |
| 6317L-G | S36420 | 7.33 | 1.65 | 4.44 | paratype | |
| 6317L-H | S36420 | 8.29 | 2.55 | 3.25 | paratype | |
| 6317L-I | S36420 | 7.89 | 1.82 | 4.34 | paratype | |
| 6317L-J | S36420 | 5.56 | 1.57 | 3.54 | paratype | |
| 6317L-K | S36420 | 7.2 | 1.78 | 4.04 | paratype | |
| 6317L-L | S36420 | 5.4 | 1.51 | 3.58 | paratype | |
| 6317L-M | S36420 | 5.93 | 2.09 | 2.84 | paratype | |
| 6317L-N | S36420 | 6.9 | 1.74 | 3.97 | paratype | |
| 6317L-O | S36420 | 6.7 | 1.8 | 3.72 | paratype | |
| 6317L-P | S36420 | 7.78 | 2.11 | 3.69 | paratype | |
| 6317L-Q | S36420 | 7.68 | 2.25 | 3.41 | paratype | |
| 6317L-R | S36420 | 5.15 | 1.45 | 3.55 | paratype | |
| 6317L-S | S36420 | 5.63 | 1.93 | 2.92 | paratype | |
| 6317L-T | S36420 | 6.47 | 1.6 | 4.04 | paratype | |
| 6317L-U | S36420 | 3.45 | 1.22 | 2.83 | paratype | |
| *n*=21 | average | 7.41 | 2.06 | 3.62 | | |
| | standard deviation | 1.89 | 0.53 | 0.47 | | |

**Supplementary Table ST3: CheckM statistics for various *V. teredinicola* gill bacterium genome assemblies and other related bacteria.**

| Genome assembly | Percent Completeness | Percent Contamination | Percent strain heterogeneity | %GC content | Contigs | Genome size (bp) | N50 (bp) |
|---|---|---|---|---|---|---|---|
| *Vadumodiolus teredinicola* (MAG) by metaBAT2 binning | 55.09 | 0.13 | 0 | 39.11 | 237 | 628,036 | 2,629 |
| *Vadumodiolus teredinicola* (MAG) by BLAST searches | 87.80 | 2.78 | 20 | 38.43 | 588 | 1,008,987 | 2,301 |
| ***Vadumodiolus teredinicola* (MAG) by combining bin and BLAST searches (final draft assembly)** | **87.88** | **2.82** | **16.67** | **38.42** | **611** | **1,060,823** | **2,316** |
| *Bathymodiolus azoricus* BazSymAV2 GCF_001298715.1 | 97.68 | 0 | 0 | 37.57 | 515 | 1,859,850 | 5,874 |
| *Bathymodiolus septemdierum* Mjoyin Knoll AP013042 | 98.68 | 0 | 0 | 38.74 | Closed-circular | 1,469,434 | Not applicable |
| *Bathymodiolus thermophilus* EPRN9N CP024634 | 97.19 | 1.99 | 100 | 38.56 | Closed-circular | 2,832,685 | Not applicable |
| *Ca*. Ruthia magnifica CP000488 | 94.16 | 0 | 0 | 34.03 | Closed-circular | 1,160,782 | Not applicable |
| *Ca*. Vesicomyosocius okutanii HA AP009247 | 93.54 | 0 | 0 | 31.59 | Closed-circular | 1,022,154 | Not applicable |
| Ca. Vesicomyosocius endoextente GCA_013416595.1 | 92.73 | 0 | 0 | 31.09 | 2 | 1,021,947 | 604,961 |
| *Ca*. Thioglobus autotrophicus EF1 CP010552 | 99.17 | 0 | 0 | 39.10 | Closed-circular | 1,512,449 | Not applicable |

**Supplementary Table ST4: Comparison between the final draft genome of *Vadumodiolus teredinicola* gill bacterium and genomes of bathymodiolin thioautotrophic symbionts.**

| Properties | *Vadumodiolus teredinicola* (by binning and BLAST) | *Bathymodiolus azoricus* BazSymAV2 | *Bathymodiolus septemdierum* Mjoyin Knoll | *Bathymodiolus thermophilus* EPRN9N |
|---|---|---|---|---|
| **NCBI Assembly Accession #** | JASISB000000000 | GCF_001298715.1 | AP013042 | CP024634 |
| **Genome size** | 1,060,823 | 1,859,850 | 1,469,434 | 2,832,685 |
| **Contigs** | 611 | 515 | Closed-circular | Closed-circular |
| **Percent completeness** | 87.88% | 97.68% | 98.68% | 97.19% |
| **Percent contamination** | 2.82% | 0.00% | 0.00% | 1.99% |
| **Strain heterogeneity** | 16.67% | 0.00% | 0.00% | 100% |
| **%GC** | 38.42% | 37.57% | 38.74% | 38.56% |
| **% coding density** | 86.07% | 88.89% | 93.65% | 89.37% |
| **Total number of genes** | 966 | 1,946 | 1,561 | 2,172 |
| **Protein coding genes (CDS)** | 948 | 1,906 | 1,523 | 2,067 |
| **rRNA genes** | 2 (5S not detected, 16S, 23S) | 3 (5S, 16S, 23S) | 3 (5S, 16S, 23S) | 3 (5S, 16S, 23S) |
| **Sulfide-quinone oxidoreductase (sqr)** | yes | no | yes | yes |
| **Adenylyl-sulfate reductase (alpha and beta subunits)** | alpha subunit only | alpha and beta subunits | alpha and beta subunits | alpha and beta subunits |
| **Ribulose bisphosphate carboxylase/oxygenase (RuBisCO)** | small and large subunits | small and large subunits | large subunit only | small and large subunits |
| **Hydrogenase (*hupL*)** | no | yes | yes | yes |
| **Nitrogenase (*nifH*)** | no | no | no | no |

**Supplementary Table ST5: GenBank accession records of bathymodiolin symbionts and related bacteria that were used in Figures 7 and Supplementary Figure SF4**

| **Genome** | **NCBI Genome assembly accession** |
|---|---|
| ***Vadumodiolus teredinicola* gill bacterium (MAG) combined bin and BLAST searches (final draft assembly)** | JASISB000000000 |
| *Bathymodiolus azoricus* BazSymAV2 | GCF_001298715.1 |
| *Bathymodiolus septemdierum* Mjoyin Knoll | AP013042 |
| *Bathymodiolus thermophilus* EPRN9N | CP024634 |
| *Ca*. Ruthia magnifica | CP000488 |
| *Ca*. Vesicomyosocius okutanii HA | AP009247 |
| *Ca.* Vesicomyosocius SY067_SCS001 | CP054877 |
| Ca. Vesicomyosocius endoextente | GCA_013416595.1 |
| *Ca*. Thioglobus autotrophicus EF1 | CP010552 |
| *Ca.* Thiodiazotropha lotti MAGLRAP2 | GCA_016842125.1 |
| *Ca.* Thiodiazotropha endoloripes | GCA_001709035.1 |
| *Ca.* Thiodiazotropha taylori MAG | GCA_016842995.1 |
| *Ca.* Thiodiazotropha Codakia orbicularis | GCA_018619755.1 |
| *Ca.* Thiodiazotropha endolucinida COS | GCA_001715975.1 |
| *Ca.* Thiodiazotropha endolucinida | GCA_016842515.1 |
| *Ca.* Endoriftia persephone strain Guaymas | GCA_013523235.1 |
| *Ca.* Endoriftia persephone strain Hot96 | GCA_000168735.1 |
| *Sedimenticola selenatireducens* DSM 17993 | GCA_000428045.1 |
| *Sedimenticola thiotaurani* SIP-G1 | CP011412 |
| *Ca.* Sedimenticola endophacoides | GCA_003062205.1 |
| *Chrysomallon squamiferum* (scaly-foot snail) endosymbiont | AP012978 |
| *Kuphus polythalamius* endosymbiont *Thiosocius teredinicola* 2141T | CP019936 |
| *Solemya pervernicosa* gill symbiont WH SV | GCF_002021235.1 |
| *Thiocapsa marina* 5811 | GCA_000223985.2 |
| *Thiocapsa bogorovii* BBS | GCA_021228795.1 |
| *Allochromatium vinosum* DSM 180 | GCA_000025485.1 |
| *Allochromatium tepidum* NZ | GCA_018409545.1 |
| *Marichromatium purpuratum* 984 | GCA_000224005.3 |
| *Marichromatium gracile* DSM 203 | GCA_004343155.1 |
| *Solemya pervernicosa* gill symbiont Sp-SM6 | GCA_002020875.1 |
| *Osedax* symbiont Rs1 | GCA_000416275.1 |
| *Thiothrix nivea* DSM 5205 | GCA_000260135.1 |
| *Thiomicrorhabdus* sp. Kp2 | GCF_000478585.1 |
| *Thiomicrorhabdus* sp. Milos-T2 | GCF_000702325.1 |
| *Thiomicrorhabdus arctica* DSM 13458 | GCF_000381085.1 |
| *Thiomicrorhabdus chilensis* DSM 12352 | GCF_000483485.1 |
| *Hydrogenovibrio kuenenii* DSM 12350 | GCF_000526715.1 |
| *Hydrogenovibrio halophilus* DSM 15072 | GCF_000384235.1 |
| *Thioalkalimicrobium aerophilum* AL3 | CP007030 |
| *Thioalkalimicrobium cyclicum* ALM1 | CP002776 |
| *Desulfurobacterium thermolithotrophum* DSM 11699 | GCA_000191045 |
